# Knowledge, practice patterns, and guideline implementation for pre-eclampsia risk management among obstetricians in China: an exploratory cross-sectional survey

**DOI:** 10.3389/fpubh.2026.1890291

**Published:** 2026-07-29

**Authors:** Sha Diao, Yuanxiu Wei, Yuan Feng, Jiayi Yang, Dan Liu, Xuefeng Jiao, Linan Zeng, Rong Zhou, Lingli Zhang

**Affiliations:** 1 Clinical Epidemiology and Evidence-Based Medicine Center, West China Hospital, Sichuan University, Chengdu, China; 2 Department of Pharmacy/Evidence-Based Pharmacy Center, West China Second University Hospital, Sichuan University, Chengdu, China; 3 Children's Medicine Key Laboratory of Sichuan Province, Chengdu, China; 4 Key Laboratory of Birth Defects and Related Diseases of Women and Children, Sichuan University, Ministry of Education, Chengdu, China; 5 West China School of Pharmacy, Sichuan University, Chengdu, China; 6 West China Biomedical Big Data Center, West China Hospital, Sichuan University, Chengdu, China; 7 Department of Obstetrics and Gynecology, West China Second University Hospital, Sichuan University, Chengdu, China; 8 Chinese Evidence-Based Medicine Center, West China Hospital, Sichuan University, Chengdu, China

**Keywords:** aspirin, China, online survey, pre-eclampsia, risk management

## Abstract

**Background:**

Pre-eclampsia remains a leading cause of maternal morbidity and mortality worldwide. However, evidence regarding the implementation of risk management strategies in low- and middle-income settings remains limited. This exploratory survey assessed knowledge, clinical practices, guideline implementation, and perceived barriers related to pre-eclampsia risk management among obstetricians in China.

**Methods:**

A web-based exploratory cross-sectional survey using convenience and snowball sampling was conducted among obstetricians in China. An anonymous 38-item questionnaire was developed based on two-step pre-eclampsia risk management strategies and distributed through academic networks and professional groups. Descriptive analyses were performed using frequencies and proportions for categorical variables and medians with interquartile ranges for continuous variables. Exploratory subgroup analyses were conducted by participant characteristics. Guideline adherence was assessed against key recommendations from four guidelines used in China.

**Results:**

Among 307 obstetricians, 91.9% reported performing pre-eclampsia risk prediction, primarily based on medical history, obstetric history, and current pregnancy factors. However, considerable variation existed in assessment timing and risk classification approaches. Although the FMF competing-risk model was recognized as important, only 61.6% had heard of the model and 36.5% reported clinical use. Limited biomarker availability and lack of automated calculation tools were the main barriers. Low-dose aspirin prophylaxis was widely reported, with 90.9% prescribing aspirin for women at moderate or high risk. The most common regimen was 100 mg once daily, initiated at 12 weeks and discontinued at 36 weeks. Only 56.0% reported initiating aspirin before 16 weeks, and 1.0% reported continuation until delivery, indicating substantial variation in aspirin management.

**Conclusions:**

This exploratory survey suggests that obstetricians in China have incorporated some components of pre-eclampsia risk management into clinical practice, but substantial variability remains in guideline implementation. Limited adoption of standardized risk assessment tools and inconsistent aspirin prophylaxis strategies highlight potential areas for improvement. Enhancing implementation support and improving access to evidence-based tools may facilitate more consistent pre-eclampsia prevention practices.

## 1 Introduction

The United Nations Sustainable Development Goals include targets to reduce global maternal mortality and prevent avoidable deaths among newborns. Pre-eclampsia, a major contributor to maternal morbidity and mortality, is associated with approximately 46,000 maternal deaths and 500,000 fetal and newborn deaths annually (1, 2). Fortunately, risk reduction strategies, including a two-step risk management approach, may help identify women at increased risk and prevent or delay the onset of pre-eclampsia. This approach involves two sequential steps. First, women at increased risk of pre-eclampsia are identified using either traditional clinical risk factors or multivariable prediction models. Second, low-dose aspirin is prescribed to reduce the risk or delay the onset of pre-eclampsia (3–8).

However, recommendations for pre-eclampsia risk management remain inconsistent across existing guidelines, particularly regarding risk factors (9), prediction approaches (10) and aspirin dosing strategies (11, 12). Such inconsistencies may contribute to variations in clinical practice among obstetricians and potentially affect adherence to guidelines recommendations. Although several studies have evaluated healthcare providers' knowledge and practices regarding pre-eclampsia risk reduction (13–17), evidence remains limited, particularly in low- and middle-income countries. Specifically, limited evidence is available regarding how pre-eclampsia risk management recommendations are implemented in Chinese clinical settings, including adherence to guideline-based risk assessment strategies, clinical uptake of the FMF competing-risks model, aspirin prophylaxis practices, and barriers affecting implementation.

Therefore, this study aimed to investigate knowledge, practice patterns, guideline implementation, and perceived barriers related to pre-eclampsia risk management among surveyed obstetricians recruited in China.

## 2 Methods

Ethical approval for this study was obtained from West China Second University Hospital, Sichuan University, China on October 29, 2024 (Approval No. 2024-345). The study followed the Strengthening the Reporting of Observational Studies in Epidemiology (STROBE) statement (18) and incorporated key recommendations from the Checklist for Reporting Results of Internet E-Surveys (CHERRIES) (19).

### 2.1 Questionnaire development

A web-based, self-administered questionnaire was developed without collecting any personally identifiable information (e.g., names, WeChat usernames, or contact details). The questionnaire was designed based on a two-step risk management framework for pre-eclampsia and consisted of 38 items organized into three sections.

The initial draft was developed by the research team based on relevant clinical guidelines and published literature. To ensure content validity, the questionnaire underwent expert review by three specialists in obstetrics. All experts held senior professional titles and doctoral degrees in obstetrics, with more than 10 years of clinical experience in the diagnosis and management of high-risk pregnancies, particularly hypertensive disorders of pregnancy and pre-eclampsia. Among them, one expert was the director of an obstetrics department and held leadership positions in national professional societies related to obstetrics and perinatal medicine. The expert panel evaluated the questionnaire regarding scientific validity, logical consistency, completeness, and clinical applicability. Based on expert feedback, the questionnaire was revised item by item to improve the accuracy and comprehensiveness of the content. For example, experts suggested adding questions regarding aspirin discontinuation or modification during pregnancy and the corresponding reasons, as well as distinguishing aspirin dosage recommendations between singleton and twin pregnancies to better reflect variations in clinical practice.

After expert review, the revised questionnaire was pilot-tested among 10 obstetricians undergoing advanced clinical training in our department to assess feasibility, comprehensibility, and response burden. The pilot participants evaluated whether the items were clearly worded, easily understood, and appropriate for completion in routine practice. Based on their feedback, further modifications were made to improve the clarity and usability of the questionnaire. For example, ambiguous wording was refined, abbreviations were replaced with their full terms, and commonly encountered autoimmune diseases (e.g., antiphospholipid syndrome and systemic lupus erythematosus) were explicitly specified to improve clarity and consistency in interpretation among respondents. Pilot participants were not included in the final analytical sample.

The first section included seven items capturing participants' demographic characteristics, such as age, gender, and educational level, including three fill-in items and four single-choice items. The second section comprised 15 items addressing current practices and perceived barriers related to pre-eclampsia risk prediction, including one fill-in item, six single-choice items, and eight multiple-choice items. This section also assessed the clinical use of the Fetal Medicine Foundation (FMF) competing-risks model, which provides individualized risk estimates based on maternal characteristics, medical history, and biomarkers (20). The predictive performance of the FMF model has been validated and shown to be effective and reproducible for first-trimester prediction of pre-eclampsia (21). In this section, risk factors were categorized into five subgroups: demographics, past medical history, past obstetric history, family history, and current pregnancy (9). The third section included 16 items on aspirin use and other prophylactic interventions, covering indications, dosage, timing, frequency, intended duration of use, and potential treatment modification, including one fill-in item, 13 single-choice items, and two multiple-choice items. These items aimed to characterize clinicians' prescribing patterns, including variations in aspirin management before the intended end point due to clinical considerations (Supplementary material).

To enhance data quality and response consistency, logical skip patterns were applied so that participants were only presented with relevant questions. Predefined ranges were set for numerical items to minimize input errors, and all items were configured as mandatory to reduce missing data. Participants were allowed to return to previous pages and modify their responses before final submission. Completeness checks were implemented before questionnaire submission to ensure that all required items were completed.

### 2.2 Participant recruitment

A cross-sectional study was conducted using a voluntary, anonymous, and incentive-free web-based survey among obstetricians in China from November 2024 to December 2024. Eligible participants were practicing obstetricians in China with experience in pre-eclampsia risk management. Participants were required to have independently provided outpatient obstetric care and participated in the assessment or management of women at risk of or with pre-eclampsia. Obstetric residents were excluded.

Given the exploratory nature of this survey and the lack of reliable prior data regarding pre-eclampsia risk management practices among obstetricians in China, the sample size was estimated based on the questionnaire item-to-response ratio, with a predefined ratio of 5:1 (22). Accordingly, a minimum of 190 participants was required. Considering that this study was one of the first investigations focusing on healthcare professionals in this specific field in China, relevant baseline data were lacking and the target population was not well enumerated. In addition, the overall numbers of eligible healthcare professionals and institutions were unclear, making it difficult to establish a complete sampling frame for probability-based sampling. Therefore, considering the exploratory nature of the study and feasibility constraints, a non-probability sampling approach was adopted (23). Convenience and snowball sampling techniques were combined for participant recruitment. Convenience sampling was conducted through existing academic collaboration networks and professional WeChat groups related to obstetrics. WeChat is one of the most widely used social networking platforms in China. Initial dissemination of the survey invitation was facilitated through the professional networks of the three senior obstetric experts involved in questionnaire review, including their relevant academic and clinical networks. These networks involved obstetric clinicians from different regions and healthcare institutions across China. Participants were further encouraged to share the survey link with eligible obstetric colleagues within their professional networks, such as collaborators and classmates, thereby facilitating snowball recruitment.

A generic web link to the questionnaire was distributed. Upon accessing the link, participants were provided with an introduction to the study and a confidentiality statement, and informed consent was obtained before participation.

The collected data were stored securely and were accessible only to the research team via password-protected systems. Potential duplicate submissions were controlled using IP address identification, with each IP address considered as a unique response source during data collection and data cleaning.

### 2.3 Data analysis

Data cleaning and analysis were performed using Microsoft^®^ Excel^®^ 2016MSO and R software (version 4.4.1; R Foundation for Statistical Computing, Vienna, Austria). Categorical variables were summarized using frequencies and percentages. Normality of continuous variables was assessed using the Shapiro–Wilk test. Since continuous variables showed non-normal distributions (all *P* < 0.05), they were summarized using medians and interquartile ranges. To enhance interpretability, different visualization methods were selected according to the structure and dimensionality of the data. Specifically, a sunburst chart was used to represent the hierarchical structure of risk factor categories and their corresponding individual factors, allowing simultaneous comparison of category-level and item-level utilization. A Sankey diagram was used to illustrate the combinations of aspirin prescribing components (dosage, frequency, timing of initiation, and cessation), enabling clear visualization of complex, multi-step clinical decision patterns.

Adherence to pre-eclampsia risk management recommendations was evaluated against four clinical guidelines (8, 24–26) published between 2019 and 2022 and currently used in China, three of which were developed by the Chinese Medical Association (CMA) (8, 24, 25). CMA is one of the non-profit national academic organizations and an important social force in the development of medical science and technology in China. Multiple guidelines were considered because no single guideline comprehensively covered all components evaluated in this study. Key recommendations related to risk prediction and prophylactic treatment were extracted as reference criteria for assessing reported clinical practices. When the same recommendation differed across guidelines, the most recently published recommendation was used as the reference standard. Each reported practice was coded as adherent or non-adherent according to whether it was consistent with the selected recommendation.

In addition, exploratory subgroup analyses were conducted to compare practices across key characteristics, including geographic region, hospital level, age, education level, professional title, and years of experience. Participants were categorized into western and non-western regions (based on economic development levels), tertiary vs. secondary or lower hospitals (reflecting differences in healthcare resources and service capacity), education level (master's degree or above vs. bachelor's degree or below), professional title (senior vs. intermediate or below), age (< 40 vs. ≥40 years), and years of experience (< 10 vs. ≥10 years). The cut-off values for age (40 years) and years of experience (10 years) were determined based on the median values of the study population. Categorization into multiple groups based on quartiles was not considered because further stratification could result in small sample sizes within certain subgroups and reduce the stability of exploratory comparisons. In addition, as no universally accepted or clinically meaningful thresholds were available for these characteristics, median-based categorization was adopted to provide an objective and consistent approach for subgroup analyses. Differences between groups were assessed using the chi-square test or Fisher's exact test, as appropriate, with all tests being two-sided and a *P* value < 0.05 considered statistically significant.

These analyses were intended to describe variations across practice settings and clinician characteristics and to provide contextual insights for interpretation, rather than to identify independent determinants; therefore, multivariable analysis was not performed.

## 3 Results

### 3.1 Participant characteristics

Of the 308 survey responses received, one participant was excluded because they were an obstetric resident who did not meet the eligibility criteria. Finally, a total of 307 valid responses from surveyed obstetricians were included in the analysis, covering 17 provincial-level regions across four economic regions in China. The majority of respondents were from the western region (81.8%), including Sichuan, Chongqing, and Guangxi. Regarding hospital level, 78.2% of participants were from tertiary hospitals, which provide specialized care and serve as regional medical centers. The age distribution was relatively diverse, with a median age of 38 years, and the largest proportion (46.9%) aged between 30 and 39 years. Among the 307 obstetricians, 87.3% were female, and more than 80% held a bachelor's or master's degree. In terms of professional title, 35.9% were chief or associate chief physicians, while 43.3% were attending physicians. The distribution of years of experience showed that 27.0% had 6–10 years and 19.9% had 11–15 years of experience, with a median of 12 years (Table 1).

**Table 1:** Demographic characteristics of participants.

Characteristics	Participants, *n* (%) (*N* = 307)
Economic regions	
Northeast	4 (1.3%)
Eastern	46 (15.0%)
Central	6 (1.9%)
Western	251 (81.8%)
Level of hospitals	
Level 3	240 (78.2%)
Level 2	66 (21.5%)
Others	1 (0.3%)
Age (in years)	
20–29	34 (11.1%)
30–39	144 (46.9%)
40–49	93 (30.3%)
50–59	31 (10.1%)
≥60	5 (1.6%)
Gender	
Male	39 (12.7%)
Female	268 (87.3%)
Highest level of education	
PhD	39 (12.7%)
Master	100 (32.6%)
Bachelor	159 (51.8%)
Below bachelor	9 (2.9%)
Job titles	
Senior/vice-senior	110 (35.9%)
Medior	133 (43.3%)
Junior	51 (16.6%)
No title	13 (4.2%)
Years of work experience	
0–5	58 (19.0%)
6–10	83 (27.0%)
11–15	61 (19.9%)
16–20	46 (15.0%)
21–25	25 (8.1%)
26–30	25 (8.1%)
>30	9 (2.9%)

### 3.2 Risk prediction

**Figure 1:**
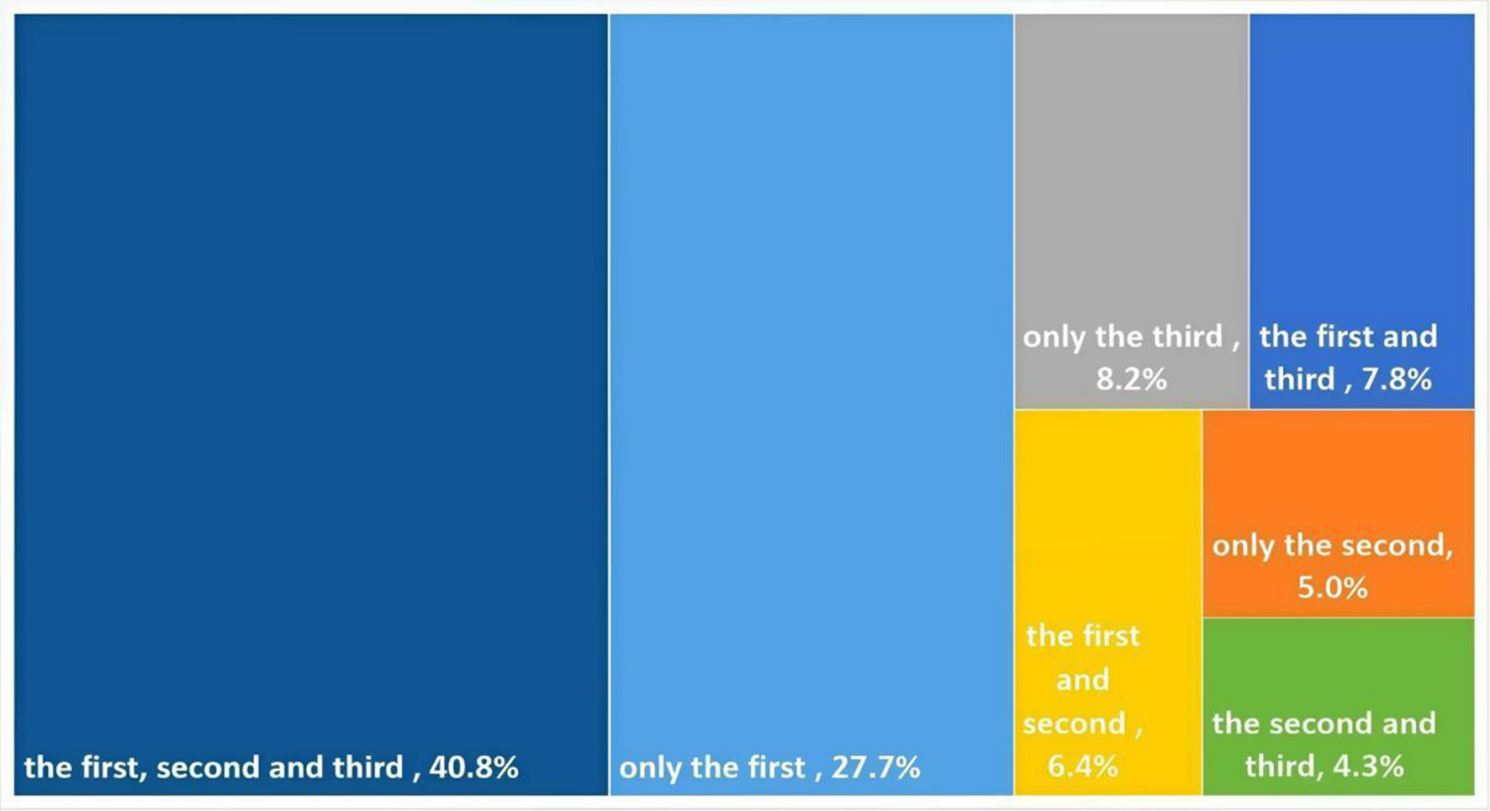
Risk prediction periods of pre-eclampsia during pregnancy. This treemap illustrates combinations of gestational periods at which risk assessment was performed. Each rectangle represents a specific combination, and its size is proportional to the percentage of participants. “first,” “second,” and “third” denote risk assessments in the first, second, and third trimester, respectively.

Almost all (91.9%) participants reported performing risk prediction for pre-eclampsia, whereas 8.1% did not. Among the 282 respondents who conducted risk prediction, 82.6% performed assessments in the first trimester, followed by 56.4% in the second trimester and 61.0% in the third trimester. Only 40.8% reported performing risk assessments across all three trimesters (Figure 1). Exploratory subgroup analyses suggested differences in risk prediction practices across participant characteristics. Obstetricians aged ≥40 years (97.7% vs. 87.6%), those with senior professional titles (96.4% vs. 89.3%), and those with ≥10 years of experience (96.4% vs. 83.6%) had higher reported rates of performing risk prediction (all *P* < 0.05). In terms of timing, first-trimester risk prediction was more frequently reported among participants from tertiary hospitals (88.0% vs. 64.6%), those with a master's degree or above (87.8% vs. 78.6%), those with senior professional titles (91.5% vs. 77.3%), and those with ≥10 years of experience (87.9% vs. 71.7%; all *P* < 0.05). By contrast, obstetricians with a bachelor's degree or below more often reported performing risk prediction in the second (62.9% vs. 48.0%) and third trimesters (69.8% vs. 49.6%), and those from western regions more frequently reported conducting risk prediction in the third trimester (64.4% vs. 44.9%; all *P* < 0.05). The detailed subgroup results were presented in Supplementary Tables S1 – S6.

**Figure 2:**
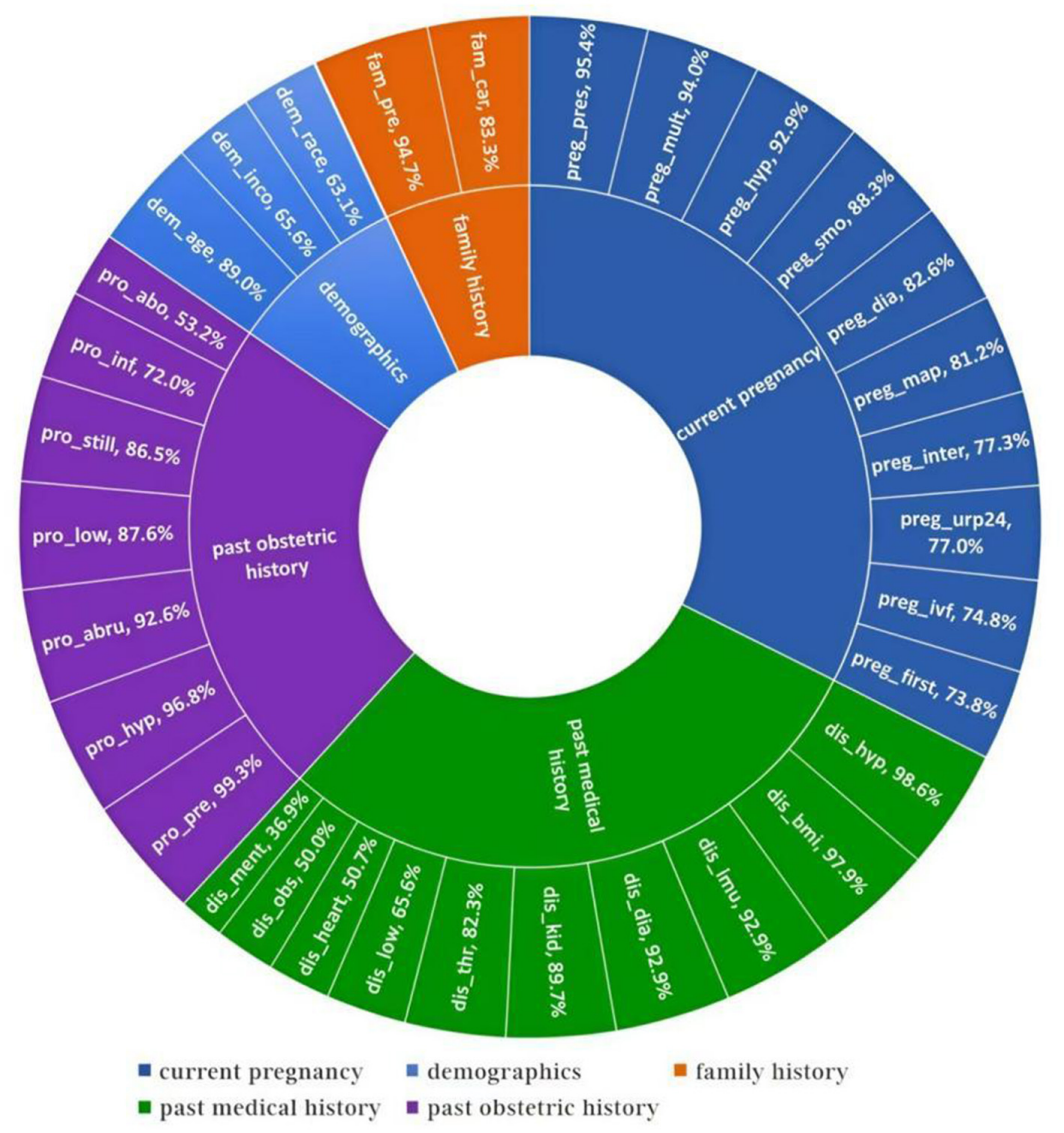
The utilization frequency of risk factors considered for pre-eclampsia risk prediction. This sunburst chart illustrates the reported utilization frequency of risk factors for risk prediction. The inner ring represents five major risk-factor categories (demographics, family history, current pregnancy, past medical history, past obstetric history), whereas the outer ring represents specific risk factors within each category. The size of each segment is proportional to the percentage of respondents reporting use of that factor. Abbreviations are used in the figure to improve readability, and full names of all abbreviated factors are provided in the legend. In demographics, dem_age, dem_inco, and dem_race refer to age, economic income, and race, respectively. In family history, fam_pre and fam_car refer to family history of pre-eclampsia and cardiovascular disease, respectively. In current pregnancy, only the top 10 most frequently used risk factors are presented, including preg_pres (increased blood pressure in early pregnancy), preg_mult (multiple pregnancy), preg_hyp (hypertensive disorders of pregnancy), preg_smo (smoking), preg_dia (gestational diabetes mellitus), preg_map (mean arterial pressure), preg_inter (interpregnancy interval ≥10 years), preg_urp24 (24-hour urine protein), preg_ivf (assisted reproductive technology), and preg_first (first pregnancy). In past medical history, dis_hyp, dis_bmi, dis_imu, dis_dia, dis_kid, dis_thr, dis_low, dis_heart, dis_obs, and dis_ment refer to chronic hypertension, overweight/obesity, autoimmune diseases, diabetes, renal disease, thrombogenic tendency, low birth weight or preterm birth, congenital heart disease, obstructive sleep apnea, and psychiatric disorders, respectively. In past obstetric history, pro_pre, pro_hyp, pro_abru, pro_low, pro_still, pro_inf, and pro_abo refer to history of pre-eclampsia/eclampsia, hypertensive disorders of pregnancy, placental abruption, small for gestational age/low birth weight infant, stillbirth, preterm birth, and miscarriage, respectively.

Regarding the use of risk factors, most obstetricians reported high utilization across all categories. Specifically, 89.7% of 282 participants used demographic characteristics, 99.3% used past medical or past obstetric history, 94.7% considered family history, and 98.2% included current pregnancy factors, with 84.0% reporting use of all five categories. Within these five categories, the most commonly reported factors were maternal age, chronic hypertension history, prior pre-eclampsia, family history of pre-eclampsia, and increased blood pressure in early pregnancy, respectively (Figure 2). In addition, some respondents reported considering other factors beyond those listed in the questionnaire. These factors were categorized into several domains, including maternal characteristics and comorbidities (education level, hypothyroidism, and intrahepatic cholestasis of pregnancy), family history-related factors (family history of venous thrombosis, diabetes, cancer, and immune diseases), lifestyle and pregnancy-related factors (alcohol consumption, calcium supplementation during pregnancy, and maternal weight gain trajectory), and ultrasound-related indicators (crown–rump length, uterine artery blood flow, uterine artery Doppler indices, and ultrasound blood flow characteristics).. Exploratory subgroup analyses suggested differences in the reported use of risk factor categories across participant characteristics. Obstetricians from non-western regions (98.0% vs. 88.0%) and those working in tertiary hospitals (91.7% vs. 83.1%) had higher reported use of demographic characteristics (all *P* < 0.05). In contrast, those from secondary or lower-level hospitals (100.0% vs. 93.1%) and those with a bachelor's degree or below (97.5% vs. 91.1%) more frequently reported considering family history (all *P* < 0.05).

For risk classification, 71.3% of obstetricians classified women into three categories (low, medium, and high risk), while 28.7% used a binary classification (low vs. high risk). No significant differences were observed across subgroups (all *P* > 0.05).

**Figure 3:**
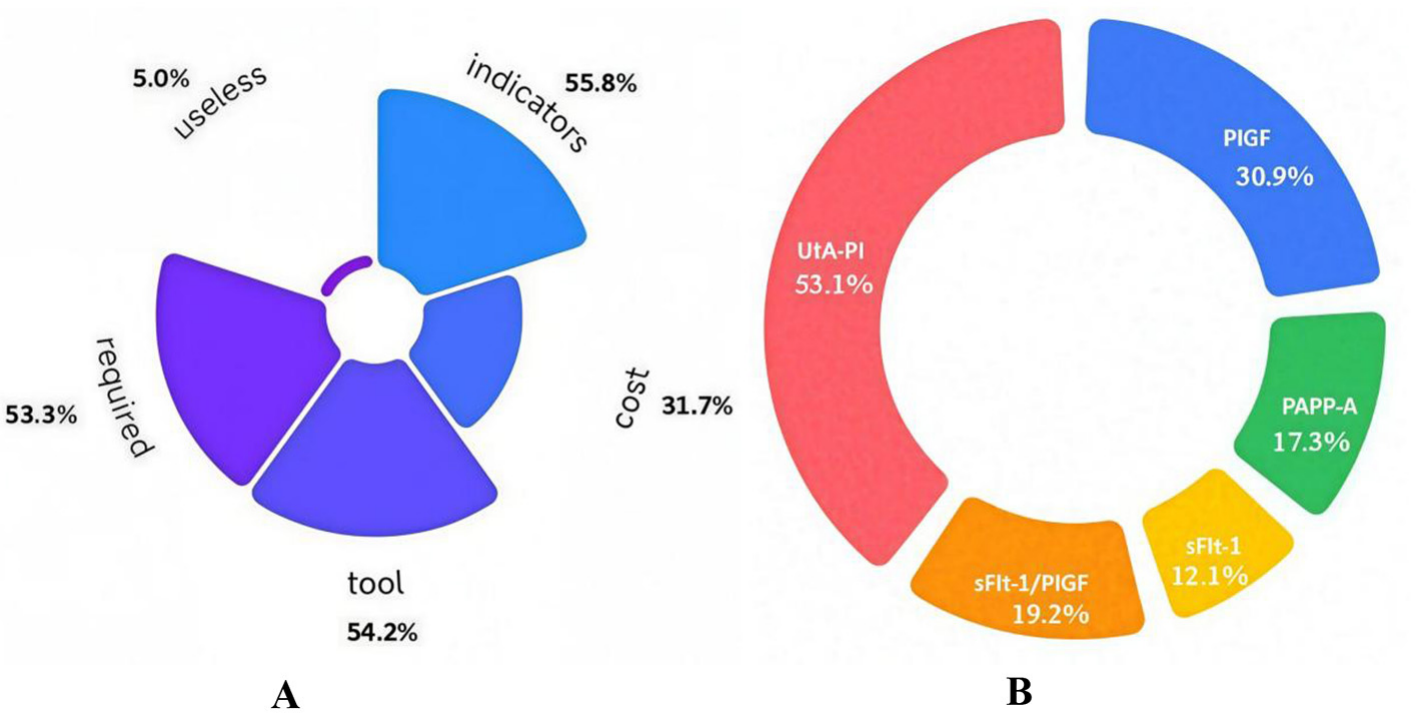
Obstacles leading to poor clinical applicability of the FMF model. **(A)** Reasons for not using the FMF mode. **(B)** Accessibility for indicators in the FMF model. These donut charts illustrate the distribution of reported barriers [Panel **(A)**] and the availability of recommended biomarkers [Panel **(B)**]. The size of each segment is proportional to the percentage of participants. PlGF, placental growth factor; PAPP-A, pregnancy associated plasma protein A; sFlt-1, soluble fms-like tyrosine kinase 1; UtA-PI, uterine artery pulsatility index.

Although the FMF competing-risks model is recommended for identifying high-risk groups by CMA (8), its clinical application appeared to be limited among surveyed obstetricians in China. While 95% of respondents acknowledged its importance, only 61.6% had heard of the model. Among those familiar with the FMF model, only 36.5% reported using it in clinical practice, with a median duration of use of 2 years. Exploratory subgroup analyses suggested differences in awareness of the FMF model across participant characteristics. Awareness of the FMF model was higher among respondents from non-western regions (73.2% vs. 59.0%), those age ≥40 years (69.8% vs. 55.6%), and those with ≥10 years of experience (66.0% vs. 53.6%; all *P* < 0.05). The main barriers to implementation were limited accessibility to required biomarkers and lack of automated calculation tools, reported by 55.8% and 54.2% of participants, respectively (Figure 3). In addition, 53.3% reported that the use of the model was not mandated, and 31.7% indicated that testing cost was too high for patients (Figure 3). Exploratory subgroup analyses suggested differences in reported barriers across participant characteristics. Limited accessibility to required biomarkers was more frequently reported by respondents with a bachelor's degree or below (64.5% vs. 46.6%) and those with intermediate or lower professional titles (63.8% vs. 45.1%; all *P* < 0.05). The perception that FMF model use was not mandated was more frequently reported by respondents with a bachelor's degree or below (62.9% vs. 43.0%), those from secondary or lower-level hospitals (80.0% vs. 46.3%), and those from western regions (57.6% vs. 33.3%; all *P* < 0.05). Cost-related barriers were more frequently reported by respondents from secondary or lower-level hospitals (56.0% vs. 25.3%; *P* < 0.05).

The biomarkers recommended by the FMF model, which are placental growth factor (PlGF), pregnancy associated plasma protein A (PAPP-A), soluble fms-like tyrosine kinase 1 (sFlt-1), the ratio of sFlt-1/PlGF and uterine artery pulsatility index (UtA-PI), were reported to be available by 30.9%, 17.3%, 12.1%, 19.2%, and 53.1% of respondents, respectively (Figure 3). Limited availability of these biomarkers may contribute to the low reported use of the FMF model among surveyed obstetricians. Exploratory subgroup analyses suggested differences in reported availability across participant characteristics. Participants from non-western regions more frequently reported availability of PlGF (62.5% vs. 23.9%), sFlt-1 (30.4% vs. 8.0%), sFlt-1/PlGF ratio (53.6% vs. 11.6%), and UtA-PI (66.1% vs. 50.2%; all *P* < 0.05). In addition, all five biomarkers were more frequently reported as available in tertiary hospitals (15.4%~57.1% vs. 4.5%−38.8%) and among participants with a master's degree or above (23.7%−59.7% vs. 2.4%−47.6%; all *P* < 0.05).

### 3.3 Prophylactic treatments

**Figure 4:**
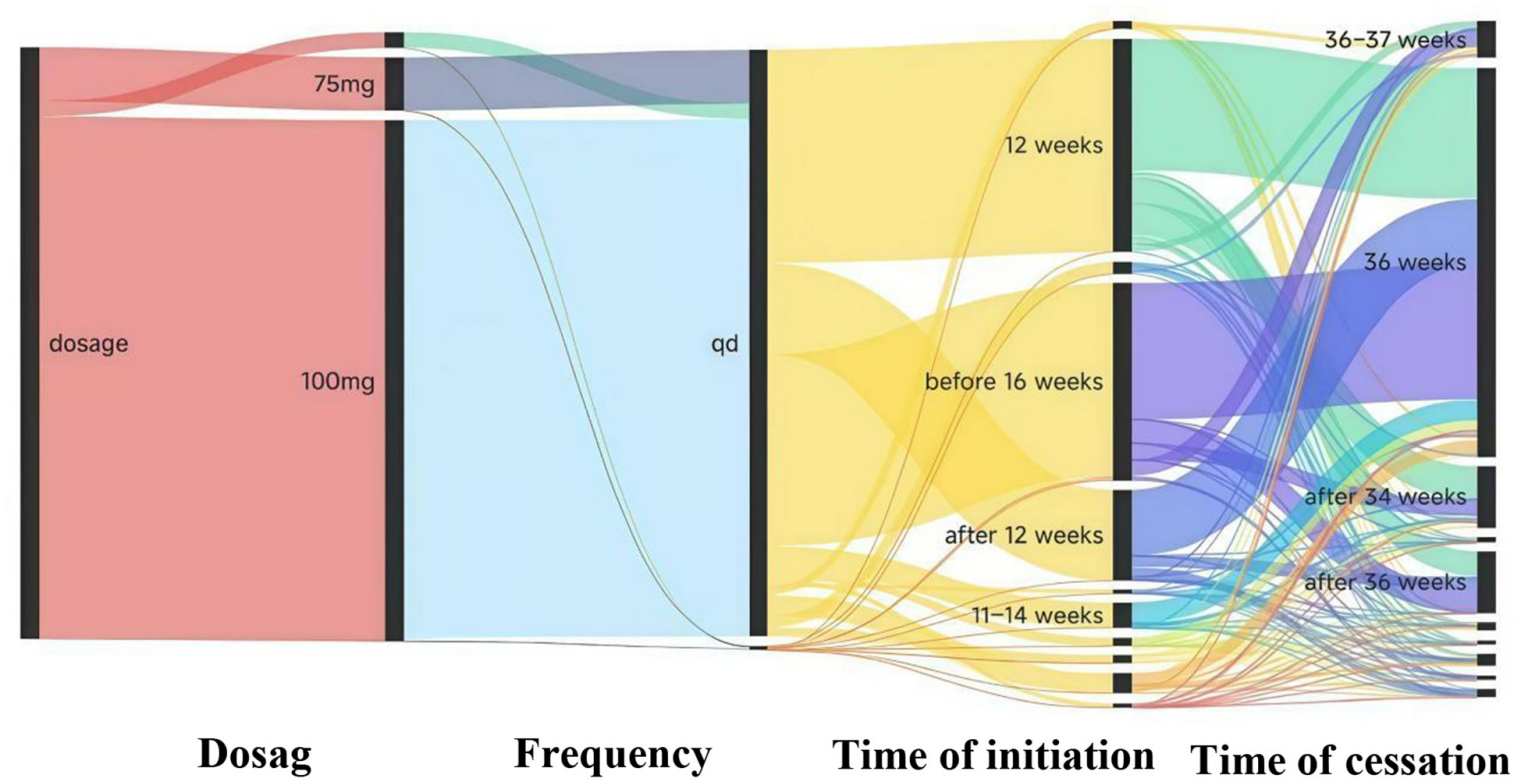
Variations in aspirin prescribing regimens among obstetricians. This Sankey diagram illustrates the combinations of key components of aspirin prescribing regimens, including dosage, frequency, time of initiation, and time of cessation. The width of each flow is proportional to the percentage of participants following each pathway.

Of all participants, 279 (90.9%) reported prescribing low-dose aspirin for women at moderate or high risk of pre-eclampsia. Among them, 51.6% reported using a uniform regimen in terms of dosage, duration, and frequency regardless of patient characteristics. Despite this, substantial variation in prescribing patterns was observed. The most commonly reported regimen was 100 mg once daily, initiated at 12 weeks of gestation and discontinued at 36 weeks (Figure 4). Exploratory subgroup analyses suggested differences in prescribing practices across participant characteristics. Obstetricians from tertiary hospitals (92.9% vs. 83.6%), those with senior professional titles (97.3% vs. 87.3%), and those with ≥10 years of experience (95.4% vs. 82.7%) had higher reported rates of prescribing aspirin (all *P* < 0.05). In addition, obstetricians from tertiary hospitals more frequently reported prescribing the commonly used 100 mg regimen (91.0% vs. 72.7%; *P* < 0.05).

In addition, 48.4% of participants reported prescribing individualized regimens based on patient characteristics. Exploratory subgroup analyses suggested differences in the use of individualized regimens across participant characteristics. Obstetricians from secondary or lower-level hospitals (60.7% vs. 45.3%) and those with < 10 years of experience (58.2% vs. 43.6%) had higher reported use of individualized regimens (all *P* < 0.05). The most frequently considered factors included BMI or body weight (84.4%), age (62.2%), hematological diseases (59.3%), multiple pregnancies (35.6%), and gestational age (31.1%). Exploratory subgroup analyses suggested differences in the reported consideration of specific factors across participant characteristics. Obstetricians from western regions more frequently reported considering multiple pregnancies (*P* < 0.05, 40.6% vs. 17.2%). In addition, obstetricians from secondary or lower-level hospitals more frequently reported considering multiple pregnancies (52.9% vs. 29.7%), gestational age (52.9% vs. 23.8%), and hematological diseases (79.4% vs. 52.5%), while those with a bachelor's degree or below more frequently reported considering gestational age (41.8% vs. 20.6%) and hematological diseases (70.1% vs. 48.5%; all *P* < 0.05).

Among the 279 obstetricians who prescribed aspirin, 19.0% reported adjusting the regimen during treatment, and 46.2% reported discontinuing aspirin prematurely. Bleeding-related factors were the most commonly reported reasons for these modifications, accounting for 62.3% of regimen adjustments and 84.5% of premature discontinuations.. These factors included coagulation dysfunction, vaginal bleeding, and gastrointestinal bleeding; however, some respondents only reported bleeding without specifying the type. Exploratory subgroup analyses suggested differences in reported regimen adjustment and discontinuation across participant characteristics. Regimen adjustment was more frequently reported by respondents with < 10 years of experience (26.4% vs. 15.4%) and those from non-western regions (30.8% vs. 16.3%), whereas premature discontinuation was more frequently reported by respondents from tertiary hospitals (49.3% vs. 33.9%) and those from non-western regions (59.6% vs. 43.2%; all *P* < 0.05). Among the 279 obstetricians who reported prescribing aspirin, 10.0% (28/279) and 5.4% (15/279) reported additionally prescribing calcium supplementation and heparin, respectively, alongside aspirin for pre-eclampsia prevention. Exploratory subgroup analyses suggested differences in reported use of combination therapies across participant characteristics. Obstetricians with senior professional titles more frequently reported prescribing calcium supplementation or heparin (23.4% vs. 10.5%) in combination with aspirin (all *P* < 0.05).

### 3.4 Adherence to guidelines

Adherence to guideline recommendations varied substantially across different components of pre-eclampsia risk management (Table 2). Among the 307 surveyed obstetricians, adherence was relatively high for the use of recommended risk factors (237, 77.2%), but remained low for performing risk assessment at all recommended time points (115, 37.5%) and for applying standardized risk classification (81, 26.4%). Similarly, adherence to the recommendation of considering the FMF model was limited (69, 22.5%). For prophylactic treatment, adherence to the recommended aspirin dosage range was high 279 (90.9%), whereas adherence to aspirin indication (156, 50.8%) and initiation timing (172, 56.0%) was moderate. In contrast, adherence to the recommendation of continuing aspirin until delivery was extremely low (3, 1.0%).

**Table 2:** Adherence to guidelines for pre-eclampsia risk management.

Recommendation	Adherence, *n* (%) (*N* = 307)
Risk prediction	
(1) Perform risk assessments at the first visit, 11–13 weeks, 19–24 weeks and 30–34 weeks during pregnancy	115 (37.5%)
(2) Perform risk assessments relied on patients' demographics, past medical history, past obstetric history, family history, and current pregnancy	237 (77.2%)
(3) Classify patients as low- and high-risk individuals regarding to risk factors	81 (26.4%)
(4) FMF model could be taken into consideration if conditions permit	69 (22.5%)
Prophylactic treatments	
(1) Low dose aspirin is recommended for high-risk women	156 (50.8%)
(2) The recommended dosage range is 50–150 mg/d	279 (90.9%)
(3) Recommended to start taking aspirin before 16 weeks of pregnancy	172 (56.0%)
(4) Recommended to take aspirin until delivery	3 (1.0%)

Non-adherence was defined as either not performing recommended actions or performing actions that do not align with guideline recommendations. Overall, 8.1% (25/307) of participants did not perform risk assessments, and 9.1% (28/307) did not prescribe aspirin. The denominator for each recommendation was determined according to the applicability of the corresponding guideline recommendation.

Exploratory subgroup analyses suggested limited differences in adherence across participant characteristics. Significant differences were observed only for aspirin dose adherence. Specifically, obstetricians from tertiary hospitals (50.8% vs. 32.8%), those with senior professional titles (56.4% vs. 41.6%), and those with ≥10 years of experience (53.8% vs. 34.5%) had higher reported adherence to the recommended dosage range (all *P* < 0.05).

## 4 Discussion

Risk prediction and aspirin treatment have been shown to be effective in preventing or delaying the onset of pre-eclampsia and are recommended by guidelines worldwide. However, inconsistencies in recommendations still exist (27), which may impede their application in clinical practice and lead to gaps in knowledge and practices among obstetricians. In this exploratory survey among obstetricians recruited in China, we identified three major findings regarding the implementation of pre-eclampsia risk management. First, surveyed obstetricians reported relatively high awareness of risk assessment strategies and widespread use of aspirin prophylaxis; however, implementation of standardized risk assessment approaches remained variable. Second, adoption of the FMF competing-risks model was limited, likely reflecting challenges related to accessibility and clinical applicability. Third, aspirin prophylaxis practices showed substantial variability, particularly regarding initiation timing and discontinuation before delivery.

Pre-eclampsia has heterogeneous clinical presentations, with disease onset occurring before 37 weeks, after 37 weeks, or even in the postpartum period (28). This variability indicates that pre-eclampsia risk is not confined to a single gestational window. Moreover, maternal characteristics and pregnancy-related factors may change throughout pregnancy. Therefore, serial risk assessments may provide opportunities to identify newly emerging risks and update individualized risk stratification. Most guidelines recommend performing risk prediction at least in the first trimester. In this survey, 82.6% of participants reported conducting risk assessment during early pregnancy, suggesting that first-trimester assessment is commonly practiced among surveyed obstetricians. However, implementation across multiple recommended time points appeared to be less consistent. The CMA recommends performing risk assessments at the first visit and at 11–13, 19–24, and 30–34 weeks of gestation (8), yet fewer than half of participants reported following this recommendation, indicating potential gaps in longitudinal risk assessment practices.

A recent expert panel identified 78 risk factors across clinical practice guidelines, covering demographics, past medical, obstetric and family history, and current pregnancy conditions (9). Notably, 84% of surveyed obstetricians reported considering all recommended categories of risk factors. The most commonly used risk factors identified in this study were consistent with those supported by existing evidence. Maternal age, family history of pre-eclampsia, and elevated blood pressure in early pregnancy have been associated with pre-eclampsia with low-, moderate-, and high-quality evidence, respectively (9). In addition, chronic hypertension history and prior pre-eclampsia have well-established associations with pre-eclampsia, supported by moderate-quality evidence (9).

The FMF competing-risk model has been shown to be effective in China (29), and is recommended for use, when conditions permit, in the latest CMA guideline (8). However, in this survey, its clinical implementation appeared to be limited among surveyed obstetricians. Although most obstetricians recognized its importance, only about one third reported using it in clinical practice. The primary barriers identified were limited accessibility, including the unavailability of required biomarkers and the lack of automated calculation tools, followed by the absence of mandatory implementation and cost-related constraints. In this study, the availability of key biomarkers recommended by the FMF model was limited, with availability ranging from 12.1% to 53.1% across different biomarkers, which may partly explain the limited uptake of the model. As these biomarkers are not routinely included in standard testing panels, the associated costs may represent an additional barrier to implementation. Similar challenges have been reported in other regions. For example, a study in the Northeastern United States found that very few providers used pre-eclampsia prediction models, partly because they have not yet been incorporated into routine standards of care (13). Together, these findings may reflect a potential mismatch between guideline recommendations and available clinical resources, particularly in settings with limited access to specialized testing and decision-support tools.

In line with other studies, most obstetricians reported prescribing aspirin for the prevention of pre-eclampsia (13, 15). However, substantial variation was observed in prescribing patterns, including dosage and duration. The most commonly prescribed regimen in our study was 100 mg once daily, initiated at 12 weeks of gestation and discontinued at 36 weeks. This differs from prescribing patterns reported in other countries. For example, 81 mg initiated at 12–16 weeks and continued until delivery is commonly used in Brazil (15), initiated at 12–14 weeks has been reported in the Northeastern United States (13), and doses of 75 mg or 81 mg are commonly prescribed in Malawi (30).

Such variation in pre-eclampsia preventive strategies, including risk prediction approaches and aspirin prophylaxis practices, may reflect not only differences in guideline recommendations but also variations in healthcare systems, prenatal care organization, and resource availability. For risk prediction, the implementation of individualized approaches integrating maternal characteristics, biomarkers, and imaging findings depends not only on clinical evidence but also on the accessibility of required tests, availability of standardized screening pathways, and feasibility within local healthcare settings. For aspirin prophylaxis, differences in recommended dosage, initiation timing, and discontinuation timing across countries may partly reflect variations in guideline frameworks, evolving evidence regarding the optimal duration of prophylaxis, and local clinical practices. For example, in settings with more comprehensive healthcare coverage, guidelines may tend to favor approaches that integrate maternal characteristics, biomarkers, and imaging findings to support more individualized risk stratification. In contrast, in settings where cost and resource constraints remain important considerations, recommendations may place greater emphasis on simplified, cost-effective approaches based on clinical risk factors and standardized aspirin regimens. Findings from this survey are consistent with this interpretation. The limited availability of FMF-related biomarkers and the associated costs may hinder the implementation of more complex risk prediction models in routine practice. Therefore, it may be important to consider context-specific, cost-effective management strategies that are feasible within local healthcare systems.

The role of individualized aspirin regimens remains controversial. In this survey, some obstetricians reported adjusting treatment strategies based on patient characteristics, such as age, BMI, multiple pregnancies, gestational age, and hematological conditions. Such individualized adjustments may reflect clinical considerations regarding differences in baseline risk and potential variations in aspirin response among pregnant women. Aspirin may contribute to pre-eclampsia prevention by irreversibly inhibiting platelet cyclooxygenase-1, thereby reducing thromboxane A2 production and platelet activation while relatively preserving prostacyclin activity (31–36). Limited studies have indicated that advancing age, obesity, inadequate doses of aspirin, and increased platelet turnover may be associated with aspirin resistance, defined as the failure of aspirin to inhibit platelet thromboxane A2 production (37–41). The optimal aspirin dosage for women with multiple pregnancies remains another area of uncertainty. Scarce evidence indicates that 160 mg or 150 mg may offer superior outcomes in twin pregnancies compared with aspirin doses ranging from 80 to 100 mg or 75 mg (42, 43). Therefore it suggests that increased aspirin dosing or frequency may be necessary in those population. In addition to aspirin, calcium supplementation is also recommended (44) and heparin may be a potential treatment (45, 46). A very small number of respondents explicitly mentioned that they would use calcium or heparin in combination in our study. However, these interventions were not examined in detail because calcium supplementation is commonly used during pregnancy and heparin use largely depends on specific clinical indications.

Notably, subgroup analyses suggested differences in reported clinical practices across participant characteristics. Obstetricians from tertiary hospitals, those with senior professional titles, and those with more clinical experience had higher reported use of standardized approaches, including aspirin use and recommended dosage regimens. These differences may reflect variations in access to clinical resources, training, and familiarity with guideline-based practices. Differences were also observed in the adoption of the FMF model. Obstetricians from non-western regions with relatively stronger economic conditions and those working in tertiary hospitals more frequently reported using the model, which may be related to better accessibility to the recommended biomarkers and supporting infrastructure. Educational level may also be associated with these patterns, as those with higher academic qualifications are more likely to work in tertiary hospitals or resource-rich settings. In addition, obstetricians with senior professional titles may be more likely to report exploring approaches beyond guideline recommendations, whereas those with intermediate or junior titles may report greater reliance on guideline-directed practices. These findings may indicate disparities in healthcare resources and implementation capacity across regions. Overall, the observed subgroup differences suggest that variability in reported pre-eclampsia management may be associated with both individual practice patterns and broader structural factors, such as healthcare setting, resource availability, and clinician experience.

Adherence to guideline recommendations varied across different components of pre-eclampsia risk management. In this survey, adherence was relatively high for risk factor assessment and aspirin dosage, but lower for recommendations related to timing of risk assessment, FMF model use, and continuation of aspirin until delivery. This pattern may reflect that recommendations requiring additional resources, structured follow-up, or system-level support could be more challenging to implement in routine clinical practice. However, the discrepancy between guideline recommendations and reported aspirin discontinuation practices should be interpreted cautiously. Although current Chinese guidelines recommend continuing low-dose aspirin until delivery, the optimal duration of aspirin prophylaxis remains an evolving topic. With accumulating evidence and ongoing clinical discussions regarding the balance between preventive benefits and potential bleeding risks during late pregnancy, earlier discontinuation of aspirin has become an increasingly considered approach in clinical practice. Therefore, the preference among surveyed obstetricians for discontinuation around 36 weeks may reflect an evolving practice pattern that has not yet been fully incorporated into current guideline recommendations. Further, the relatively high reported aspirin prescribing rate but lower adherence to guideline-defined indications may reflect differences between clinical decision-making and formal guideline-based risk classification. Clinicians may prescribe aspirin based on their own risk stratification approaches, while adherence assessment requires consistency with specific guideline-defined criteria.

Despite these findings, several limitations should be acknowledged. First, due to feasibility constraints and the lack of a complete sampling frame, a non-probability sampling strategy (convenience and snowball sampling) was adopted. This approach may introduce selection bias and limit the representativeness of the study population. Although the sample size was considered adequate for exploratory descriptive analysis based on the recommended sample-to-item ratio, it does not overcome the potential limitations associated with non-probability sampling. Furthermore, participation was voluntary, and obstetricians with greater interest in pre-eclampsia prevention or guideline-based management may have been more likely to participate, potentially influencing the reported levels of awareness and adherence. Therefore, the findings should be interpreted with caution when generalizing to all obstetricians in China. Second, the distribution of participants across regions and hospital levels was imbalanced, with a higher proportion recruited from western regions and tertiary hospitals. This may limit the applicability of the findings to obstetricians practicing in other geographic regions and healthcare settings, particularly those in lower-level hospitals. Although subgroup analyses were conducted to explore variations across participant characteristics, these analyses were descriptive in nature and were not intended to identify independent determinants. Accordingly, no adjustment for multiple comparisons was performed, and multivariable analyses were not undertaken; therefore, potential confounding factors could not be fully accounted for. Third, the data were self-reported and collected through an anonymous online survey, which may introduce information bias. However, several quality control measures were implemented, including mandatory responses, logical checks, and range restrictions, to improve data completeness and consistency, although the accuracy of responses could not be independently verified. Finally, some aspects of clinical practice were not explored in depth in order to ensure questionnaire feasibility and response completion. Future studies using more representative sampling strategies and more detailed data collection are needed to further validate and extend these findings. Overall, although the findings from this survey may not be fully generalizable, they provide important insights into reported clinical practices and challenges in the prevention and management of pre-eclampsia among surveyed obstetricians in China and may help inform future guideline implementation strategies and healthcare policy development.

## 5 Conclusions

In this exploratory survey, surveyed obstetricians recruited in China reported familiarity with two-step risk management strategies for pre-eclampsia. Recommended risk factor categories were commonly considered during risk assessment, and low-dose aspirin was widely prescribed for prevention. However, substantial variability remained in reported clinical practice patterns, particularly regarding the implementation of timing-related recommendations and the use of standardized risk assessment approaches. Differences between reported practices and guideline recommendations, especially regarding aspirin discontinuation timing, may partly reflect evolving clinical practice and the need for continued evaluation of evidence and guideline updates. These findings highlight the importance of ensuring that guideline recommendations are clinically applicable, accessible, and feasible within local healthcare settings. Effective implementation of pre-eclampsia prevention strategies may require coordinated efforts involving clinician education, evidence-based tools, resource availability, and broader healthcare system support.

## Data Availability

The raw data supporting the conclusions of this article will be made available by the authors, without undue reservation.
