# Supplementary material for: Knowledge, practice patterns, and guideline implementation for pre-eclampsia risk management among obstetricians in China: an exploratory cross-sectional survey
Source: Front Public Health. 2026 Jul 29;14:1890291. doi: 10.3389/fpubh.2026.1890291 (PMC13461581; doi:10.3389/fpubh.2026.1890291)
Supplement: Supplementary file 1 [file Table_1.docx]

Supplementary Material

**Questionnaire about the risk management behaviors on pre-eclampsia**

**among obstetricians in China**

Dear doctors,

Hello!

We are conducting a research survey on the risk management behaviors on pre-eclampsia among obstetricians, including risk prediction and prophylactic treatments.

Pre-eclampsia is a severe type of hypertensive disorder complicating pregnancy, posing a significant threat to maternal and fetal health. Accurately prediction of the risk of pre-eclampsia and taking reasonable prophylactic treatments are crucial for ensuring the safety of both mothers and infants.

The purpose of this survey is to understand your practical experiences and obstacles in risk prediction and prophylactic treatments for pre-eclampsia. It is expected to take 3-5 minutes to complete. Your responses will have a significant impact on our research. We sincerely appreciate your support and participation.

We promise that the information collected from this survey will only be used for scientific research. Your personal information and responses will be kept strictly confidential. Please fill in the questionnaire based on your actual situation. If you have any questions or suggestions, please feel free to contact us. Thank you again for your participation!

**Part I Basic Information**

1. **Name of your institution:**
2. **Level of your institution:**

A. Level 3	B. Level 2	C. Others, please specify: ______

**3. Your age: ___ years old (20~80)**

**4. Your gender:**

A. Female	B. Male

**5. Years of experience in obstetric-related clinical diagnosis and treatments:** ___ years (0~70)

**6. Your professional title:**

A. Senior (including associate senior)	B. Intermediate	C. Junior	D. None

**7. Your highest degree:**

A. Doctor’s	B. Master's	C. Bachelor's	D. Below bachelor’s

**Part II Risk Prediction for Pre-eclampsia**

8. **Do you assess patients' risk of developing pre-eclampsia during diagnosis and treatments?**

A. No (Skip to Question 17)	B. Yes

**9. At which stage(s) do you assess patients' risk of developing pre-eclampsia and how many times? (Multiple choices)**

A. Early pregnancy (before 13^+6^ weeks), total ___ times

B. Mid-pregnancy (14~27^+6^ weeks), total ___ times

C. Late pregnancy (28 weeks and beyond), total ___ times

D. Others, pregnancy at ___ weeks, total ___ times

**10. Please select the factors you use to assess patients' risk of developing pre-eclampsia. (Multiple choices)**

A. Patient's basic demographic characteristics (Skip to Question 11)

B. Patient's past medical history (Skip to Question 12)

C. Patient's past obstetric history (Skip to Question 13)

D. Patient's family history (Skip to Question 14)

E. Patient's current pregnancy status (Skip to Question 15)

F. Other patient’s conditions, please specify: ______

**11. Patient's basic demographic characteristics used for risk prediction (Multiple choices)**

A. Age	B. Race	C. Economic income	D. Others, please specify: ______

**12. Patient's past medical history used for risk prediction (Multiple choices)**

A. Overweight/obesity according to BMI	B. Chronic hypertension

C. Diabetes	D. Obstructive sleep apnea

E. Autoimmune diseases (antiphospholipid syndrome, systemic lupus erythematosus, etc.)

F. Renal disease	G. Congenital heart disease	H. Thrombogenic tendency

I. Psychiatric disorders J. Patient who was a low-birth-weight infant or premature infant

K. Others, please specify: ______

13. **Patient's past obstetric history used for risk prediction (Multiple choices)**
A. History of pre-eclampsia/eclampsia

B. History of other hypertensive disorders during pregnancy

C. History of stillbirth

D. History of placental abruption

E. History of preterm birth

F. History of small for gestational age (fetal growth restriction)/low birth weight infant

G. History of miscarriage

H. Others, please specify: ______

**14. Patient's family history used for risk prediction (Multiple choices)**

A. Family history of pre-eclampsia

B. Family history of cardiovascular diseases

C. Others, please specify: ______

**15. Patient's current pregnancy status used for risk prediction (Multiple choices)**

A. Smoking	B. The first pregnancy	C. Not delivery

D. Multiple pregnancy (twins/triplets, etc.) E. Chromosomal abnormalities in the patient

F. Fetal chromosomal abnormalities	G. Elevated blood pressure in early pregnancy

H. Other hypertensive disorders during pregnancy

I. Mean arterial pressure (MAP) level [(systolic blood pressure + 2 * diastolic blood pressure) / 3]

J. Concurrent gestational diabetes mellitus	K. Assisted reproductive technology

L. Birth interval≥10 years	M. Gestational trophoblastic disease	N. Irregular antenatal care)	O. Urinary protein misfolding levels	P. Placental growth factor (PlGF) levels

Q. Pregnancy-associated plasma protein-A (PAPP-A) levels

R. Soluble fms-like tyrosine kinase-1 (sFlt-1) levels	S. Positive urine protein

T. 24-hour urine protein quantitation	U. Urine protein/creatinine ratio

V. Uterine artery pulsatility index (UTPI) W. Uterine artery systolic/diastolic ratio (S/D)

X. Uterine artery resistance index (RI)	Y. Others, please specify: ______

**16. How do you classify patients into different risk groups for pre-eclampsia?**

A. Low-risk, high-risk B. Low-risk, medium-risk, high-risk C. Others, please specify: ______

**17. Have you heard of the pre-eclampsia risk calculator recommended by the Chinese Medical Association, developed by the Fetal Medicine Foundation (FMF)?**

A. No (Skip to Question 22)	B. Yes

**18. Do you use the risk calculator developed by FMF to assess patients' risk of developing pre-eclampsia?**

A. No (Skip to Question 21)	B. Yes

**19. Start date of use:** ___ year (1990~2024)

**20. Does your institution require the mandatory use of this tool?**

A. No	B. Yes

**21. Reasons for not using the FMF tool (Multiple choices)**

A. Recommended test indicators are not fully available in our medical institution (e.g., PlGF testing is not available, UTPI is not reported in ultrasound examinations)

B. Patients are unwilling to use because of high cost of testing related indicators

C. No tool for automatic calculation of pre-eclampsia risk

D. Medical institution does not require its use

E. Personally do not see the need

F. Others, please specify: ______

**22. Can your institution measure or report the following indicators?**
(1) Placental growth factor (PlGF)
A. No	B. Yes, at a price of approximately _____ yuan per unit	C. Not sure
(2) Pregnancy-associated plasma protein-a (PAPP-A)
A. No	B. Yes, at a price of approximately _____ yuan per unit	C. Not sure
(3) Soluble fms-like tyrosine kinase 1 (sFlt-1)
A. No	B. Yes, at a price of approximately _____ yuan per unit	C. Not sure
(4) sFlt-1/PlGF ratio
A. No	B. Yes, at a price of approximately _____ yuan per unit	C. Not sure
(5) Uterine artery pulsatility index (UTPI)
A. No	B. Yes, at a price of approximately _____ yuan per unit	C. Not sure

**Part III: Preventive Pharmaceutical Treatment**

**23. Would you prescribe aspirin to patients for the prevention of pre-eclampsia?**
A. No (Skip to Question 37)	B. Yes

**24. For which patients would you prescribe aspirin? (Multiple Choice)**
A. All patients
B. Patients judged to be at medium risk in the risk assessment
C. Patients judged to be at high risk in the risk assessment
D. Patients judged to be at medium to high risk in the risk assessment
E. Others, please specify _____

**25. Would you prescribe different regimens of aspirin (dose, frequency, duration, etc.) for patients with different risk factor characteristics?**
A. No	B. Yes (Skip to Question 30)

**26. What is the single dose of aspirin you prescribing?**

A.50mg	B. 75mg	C. 81mg	D. 100mg	E. 150mg

F. 162mg G. Others, please specify _____

**27. What is the frequency of aspirin you prescribing?**

A. Once a day B. Twice a day C. Three times a day D.Others, please specify _____
28. **What is the initiation time of aspirin you prescribing?**
A. The first antenatal care	B. 12 weeks of gestation	C. 20 weeks of gestation

D. Before 16 weeks gestation	E. After 12 weeks of gestation	F. 11 to 14 weeks of gestation	G. 11 to 15 weeks of gestation	H. 12 to 28 weeks gestation I. Before 20 weeks of gestation	J. Others, please specify _____

**29. What is the ending time of aspirin you prescribing?**

A. 36 weeks of gestation B. At the time of termination	C. After 34 weeks of gestation	D. D. After 36 weeks of gestation E. 26 to 28 weeks of gestation	F. 36 to 37 weeks of gestation	G. 34 weeks of gestation to termination H.Others, please specify _____

**30. Based on which patients’ characteristics do you prescribe different regiments of aspirin? (Multiple Choice)**
A. Age, with the cumulative aspirin dose ______ (increasing/decreasing) as age increases (Skip to Question 35)
B. BMI, with the cumulative aspirin dose ______ (increasing/decreasing) as BMI increases (Skip to Question 35)
C. Singleton/twin/multiple pregnancy
D. Gestational age, with the cumulative aspirin dose ______ (increasing/decreasing) as gestational age increases (Skip to Question 35)
E. Hematological complications (such as coagulation disorders), with the cumulative aspirin dose ______ (increasing/decreasing) if present (Skip to Question 35)
F. Others, please specify _____ (Skip to Question 35)

**31. What is the single dose of aspirin you prescribing?**

**(1) Singleton**

A. 50mg	B. 75mg	C. 81mg	D. 100mg	E. 150mg

F. 162mg	G. Others, please specify _____

**(2) Multiple pregnancy**

A. 50mg	B. 75mg	C. 81mg	D. 100mg	E. 150mg

F. 162mg	G. Others, please specify _____

**32. What is the frequency of aspirin you prescribing?**

**(1) Singleton**

A. Once a day B. Twice a day C. Three times a day D.Others, please specify _____

**(2) Multiple pregnancy**

A. Once a day B. Twice a day C. Three times a day D.Others, please specify _____

**33. What is the initiation time of aspirin you prescribing?**

**(1) Singleton**

A. The first antenatal care	B. 12 weeks of gestation	C. 20 weeks of gestation

D. Before 16 weeks gestation	E. After 12 weeks of gestation	F. 11 to 14 weeks of gestation	G. 11 to 15 weeks of gestation	H. 12 to 28 weeks gestation I. Before 20 weeks of gestation	J. Others, please specify _____

**(2) multiple pregnancy**

A. First antenatal care	B. 12 weeks of gestation	C. 20 weeks of gestation

D. Before 16 weeks gestation	E. After 12 weeks of gestation	F. 11 to 14 weeks of gestation	G. 11 to 15 weeks of gestation	H. 12 to 28 weeks gestation I. Before 20 weeks of gestation	J. Others, please specify _____

**34. What is the ending time of aspirin you prescribing?**

**(1) Singleton**

A. 36 weeks of gestation B. At the time of termination C. After 34 weeks of gestation

D. After 36 weeks of gestation E. 26 to 28 weeks of gestation	F. 36 to 37 weeks of gestation	G. 34 weeks of gestation to termination H.Other, please specify _____

**(2) Multiple pregnancy**

A. 36 weeks of gestation B. At the time of termination	C. After 34 weeks of gestation	D. After 36 weeks of gestation E. 26 to 28 weeks of gestation	F. 36 to 37 weeks of gestation	G. 34 weeks of gestation to terminate pregnancy H.Other, please specify _____

**35. Do you adjust the dosage or frequency of aspirin midway during treatments?**
A. No	B. Yes, please specify the reason _____

**36. Do you ask patients to stop taking aspirin midway during treatments?**
A. No	B. Yes, please specify the reason _____

**37. Do you prescribe other medications for the prevention of pre-eclampsia?**
A. No (End of questionnaire)	B. Yes
**38. The medications you prescribing for the prevention of pre-eclampsia (excluding aspirin) are:**
Medication 1: Name _____, single dose _____, frequency _____, initiation time_____, ending time _____

Medication 2: Name _____, single dose _____, frequency _____, initiation time_____, ending time _____

Medication 3: Name _____, single dose _____, frequency _____, initiation time_____, ending time _____

Supplementary Table

# Table S1 Knowledge, practices, and adherence to clinical practice guidelines for pre-eclampsia risk management among obstetricians by economic region (Western vs Non-western)

| **Module** | **Variable** | **Level** | **Western** | **Non-western** | ***P* value** |
| --- | --- | --- | --- | --- | --- |
| **Risk prediction** | **Risk prediction** | No | 18 (7.2%) | 7 (12.5%) | 0.184* |
|  |  | Yes | 233 (92.8%) | 49 (87.5%) |  |
|  | **Timing of prediction** | | | | |
|  | first trimester | No | 44 (18.9%) | 5 (10.2%) | 0.145 |
|  |  | Yes | 189 (81.1%) | 44 (89.8%) |  |
|  | second trimester | No | 97 (41.6%) | 26 (53.1%) | 0.142 |
|  |  | Yes | 136 (58.4%) | 23 (46.9%) |  |
|  | third trimester | No | 83 (35.6%) | 27 (55.1%) | **0.011** |
|  |  | Yes | 150 (64.4%) | 22 (44.9%) |  |
|  | all three trimesters | No | 135 (57.9%) | 32 (65.3%) | 0.340 |
|  |  | Yes | 98 (42.1%) | 17 (34.7%) |  |
|  | **Risk factors used** | | | | |
|  | demographics | No | 28 (12.0%) | 1 (2.0%) | **0.037** |
|  |  | Yes | 205 (88.0%) | 48 (98.0%) |  |
|  | past medical history | No | 2 (0.9%) | 0 (0.0%) | 1.000* |
|  |  | Yes | 231 (99.1%) | 49 (100.0%) |  |
|  | past obstetric history | No | 1 (0.4%) | 1 (2.0%) | 0.318* |
|  |  | Yes | 232 (99.6%) | 48 (98.0%) |  |
|  | family history | No | 10 (4.3%) | 5 (10.2%) | 0.150* |
|  |  | Yes | 223 (95.7%) | 44 (89.8%) |  |
|  | current pregnancy | No | 4 (1.7%) | 1 (2.0%) | 1.000* |
|  |  | Yes | 229 (98.3%) | 48 (98.0%) |  |
|  | all five categories | No | 39 (16.7%) | 6 (12.2%) | 0.435 |
|  |  | Yes | 194 (83.3%) | 3 (87.8%) |  |
|  | **Risk classification** | Binary (low/high) | 65 (27.9%) | 16 (32.7%) | 0.504 |
|  |  | Ternary (low/medium/high) | 168 (72.1%) | 33 (67.3%) |  |
|  | **Awareness of FMF model** | No | 103 (41.0%) | 15 (26.8%) | **0.047** |
|  |  | Yes | 148 (59.0%) | 41 (73.2%) |  |
|  | **Use of FMF model** | No | 99 (66.9%) | 21 (51.2%) | 0.065 |
|  |  | Yes | 49 (33.1%) | 20 (48.8%) |  |
|  | **Barriers to FMF model implementation** | | | | |
|  | limited biomarker availability | No | 44 (44.4%) | 9 (42.9%) | 0.894 |
|  |  | Yes | 55 (55.6%) | 12 (57.1%) |  |
|  | no automatic calculation tools | No | 43 (43.4%) | 12 (57.1%) | 0.252 |
|  |  | Yes | 56 (56.6%) | 9 (42.9%) |  |
|  | no mandatory | No | 42 (42.4%) | 14 (66.7%) | **0.043** |
|  |  | Yes | 57 (57.6%) | 7 (33.3%) |  |
|  | cost | No | 65 (65.7%) | 17 (81.0%) | 0.171 |
|  |  | Yes | 34 (34.3%) | 4 (19.0%) |  |
|  | no perceived benefit | No | 94 (94.9%) | 20 (95.2%) | 1.000* |
|  |  | Yes | 5 (5.1%) | 1 (4.8%) |  |
|  | **Biomarker availability** | | | | |
|  | PlGF | No/Unclear | 191 (76.1%) | 21 (37.5%) | **<0.001** |
|  |  | Yes | 60 (23.9%) | 35 (62.5%) |  |
|  | PAPP-A | No/Unclear | 211 (84.1%) | 43 (76.8%) | 0.193 |
|  |  | Yes | 40 (15.9%) | 13 (23.2%) |  |
|  | sFlt-1 | No/Unclear | 231 (92.0%) | 39 (69.6%) | **<0.001** |
|  |  | Yes | 20 (8.0%) | 17 (30.4%) |  |
|  | sFlt-1/PlGF | No/Unclear | 222 (88.4%) | 26 (46.4%) | **<0.001** |
|  |  | Yes | 29 (11.6%) | 30 (53.6%) |  |
|  | UtA-PI | No/Unclear | 125 (49.8%) | 19 (33.9%) | **0.031** |
|  |  | Yes | 126 (50.2%) | 37 (66.1%) |  |
| **Prophylactic treatments** | **Aspirin use** | No | 24 (9.6%) | 4 (7.1%) | 0.570 |
|  |  | Yes | 227 (90.4%) | 52 (92.9%) |  |
|  | **Individualized regimen** | No | 121 (53.3%) | 23 (44.2%) | 0.238 |
|  |  | Yes | 106 (46.7%) | 29 (55.8%) |  |
|  | **Aspirin Dose** | 100mg | 109 (90.1%) | 18 (78.3%) | 0.151* |
|  |  | Others | 12 (9.9%) | 5 (21.7%) |  |
|  | **Aspirin frequency** | Once daily | 120 (99.2%) | 23 (100.0%) | 1.000* |
|  |  | Other frequencies | 1 (0.8%) | 0 (0.0%) |  |
|  | **Aspirin initiation** | 12 weeks of gestation | 47 (38.8%) | 5 (21.7%) | 0.117* |
|  |  | Others | 74 (61.2%) | 18 (78.3%) |  |
|  | **Aspirin discontinuation** | 36 weeks of gestation | 79 (65.3%) | 16 (69.6%) | 0.692 |
|  |  | others | 42 (34.7%) | 7 (30.4%) |  |
|  | **Factors influencing individualized** | | | | |
|  | maternal age | No | 37 (34.9%) | 14 (48.3%) | 0.188 |
|  |  | Yes | 69 (65.1%) | 15 (51.7%) |  |
|  | BMI/weight | No | 17 (16.0%) | 4 (13.8%) | 1.000* |
|  |  | Yes | 89 (84.0%) | 25 (86.2%) |  |
|  | multiple pregnancy | No | 63 (59.4%) | 24 (82.8%) | **0.020** |
|  |  | Yes | 43 (40.6%) | 5 (17.2%) |  |
|  | gestational age | No | 71 (67.0%) | 22 (75.9%) | 0.360 |
|  |  | Yes | 35 (33%) | 7 (24.1%) |  |
|  | hematologic disorders | No | 42 (39.6%) | 13 (44.8%) | 0.613 |
|  |  | Yes | 64 (60.4%) | 16 (55.2%) |  |
|  | **Regimen modification during treatment** | No | 190 (83.7%) | 36 (69.2%) | **0.016** |
|  |  | Yes | 37 (16.3%) | 16 (30.8%) |  |
|  | **Premature discontinuation** | No | 129 (56.8%) | 21 (40.4%) | **0.032** |
|  |  | Yes | 98 (43.2%) | 31 (59.6%) |  |
|  | **Concomitant use of calcium or heparin** | No | 189 (83.3%) | 47 (90.4%) | 0.199 |
|  |  | Yes | 38 (16.7%) | 5 (9.6%) |  |
| **Adherence to guidelines** | **Timing adherence** | No | 135 (57.9%) | 32 (65.3%) | 0.340 |
|  |  | Yes | 98 (42.1%) | 17 (34.7%) |  |
|  | **Risk factor adherence** | No | 39 (16.7%) | 6 (12.2%) | 0.435 |
|  |  | Yes | 194 (83.3%) | 43 (87.8%) |  |
|  | **Risk classification adherence** | No | 168 (72.1%) | 33 (67.3%) | 0.504 |
|  |  | Yes | 65 (27.9%) | 16 (32.7%) |  |
|  | **FMF model adherence** | No | 99 (66.9%) | 21 (51.2%) | 0.065 |
|  |  | Yes | 49 (33.1%) | 20 (48.8%) |  |
|  | **Aspirin indication adherence** | No | 176 (77.5%) | 37 (71.2%) | 0.329 |
|  |  | Yes | 51 (22.5%) | 15 (28.8%) |  |
|  | **Aspirin dose adherence** | No | 130 (51.8%) | 33 (58.9%) | 0.333 |
|  |  | Yes | 121 (48.2%) | 23 (41.1%) |  |
|  | **Aspirin initiation adherence** | No | 82 (67.8%) | 14 (60.9%) | 0.520 |
|  |  | Yes | 39 (32.2%) | 9 (39.1%) |  |
|  | **Aspirin discontinuation adherence** | No | 119 (98.3%) | 23 (100.0%) | 1.000* |
|  |  | Yes | 2 (1.7%) | 0 (0.0%) |  |
| ***Note***: Data are presented as n (%). *P* values were calculated using χ² test or Fisher’s exact test (*).  *P* values in bold indicated statistically significant differences between groups (*P* < 0.05). | | | | | |

**Table S2. Knowledge, practices, and adherence to clinical practice guidelines for pre-eclampsia risk management among obstetricians by hospital level (Secondary or below vs Tertiary)**

| **Module** | **Variable** | **Level** | **Secondary or below** | **Tertiary** | ***P* value** |
| --- | --- | --- | --- | --- | --- |
| **Risk prediction** | **Risk prediction** | No | 2 (3.0%) | 23 (9.6%) | 0.081 |
|  |  | Yes | 65 (97.0%) | 217 (90.4%) |  |
|  | **Timing of prediction** | | | | |
|  | first trimester | No | 23 (35.4%) | 26 (12.0%) | **<0.001** |
|  |  | Yes | 42 (64.6%) | 191 (88.0%) |  |
|  | second trimester | No | 23 (35.4%) | 100 (46.1%) | 0.127 |
|  |  | Yes | 42 (64.6%) | 117 (53.9%) |  |
|  | third trimester | No | 19 (29.2%) | 91 (41.9%) | 0.065 |
|  |  | Yes | 46 (70.8%) | 126 (58.1%) |  |
|  | all three trimesters | No | 37 (56.9%) | 130 (59.9%) | 0.668 |
|  |  | Yes | 28 (43.1%) | 87 (40.1%) |  |
|  | **Risk factors used** | | | | |
|  | demographics | No | 11 (16.9%) | 18 (8.3%) | **0.045** |
|  |  | Yes | 54 (83.1%) | 199 (91.7%) |  |
|  | past medical history | No | 0 (0.0%) | 2 (0.9%) | 1.000* |
|  |  | Yes | 65 (100.0%) | 215 (99.1%) |  |
|  | past obstetric history | No | 0 (0.0%) | 2 (0.9%) | 1.000* |
|  |  | Yes | 65 (100.0%) | 215 (99.1%) |  |
|  | family history | No | 0 (0.0%) | 15 (6.9%) | **0.026*** |
|  |  | Yes | 65 (100.0%) | 202 (93.1%) |  |
|  | current pregnancy | No | 0 (0.0%) | 5 (2.3%) | 0.593* |
|  |  | Yes | 65 (100.0%) | 212 (97.7%) |  |
|  | all five categories | No | 11 (16.9%) | 34 (15.7%) | 0.809 |
|  |  | Yes | 54 (83.1%) | 183 (84.3%) |  |
|  | **Risk classification** | Binary (low/high) | 16 (24.6%) | 65 (30.0%) | 0.404 |
|  |  | Ternary (low/medium/high) | 49 (75.4%) | 152 (70.0%) |  |
|  | **Awareness of FMF model** | No | 28 (41.8%) | 90 (37.5%) | 0.523 |
|  |  | Yes | 39 (58.2%) | 150 (62.5%) |  |
|  | **Use of FMF model** | No | 25 (64.1%) | 95 (63.3%) | 0.929 |
|  |  | Yes | 14 (35.9%) | 55 (36.7%) |  |
|  | **Barriers to FMF model implementation** | | | | |
|  | limited biomarker availability | No | 7 (28.0%) | 46 (48.4%) | 0.067 |
|  |  | Yes | 18 (72.0%) | 49 (51.6%) |  |
|  | no automatic calculation tools | No | 9 (36.0%) | 46 (48.4%) | 0.267 |
|  |  | Yes | 16 (64.0%) | 49 (51.6%) |  |
|  | no mandatory | No | 5 (20.0%) | 51 (53.7%) | **0.003** |
|  |  | Yes | 20 (80.0%) | 44 (46.3%) |  |
|  | cost | No | 11 (44.0%) | 71 (74.7%) | **0.003** |
|  |  | Yes | 14 (56.0%) | 24 (25.3%) |  |
|  | no perceived benefit | No | 24 (96.0%) | 90 (94.7%) | 1.000* |
|  |  | Yes | 1 (4.0%) | 5 (5.3%) |  |
|  | **Biomarker availability** | | | | |
|  | PlGF | No/Unclear | 64 (95.5%) | 148 (61.7%) | **<0.001** |
|  |  | Yes | 3 (4.5%) | 92 (38.3%) |  |
|  | PAPP-A | No/Unclear | 63 (94.0%) | 191 (79.6%) | **0.006** |
|  |  | Yes | 4 (6.0%) | 49 (20.4%) |  |
|  | sFlt-1 | No/Unclear | 67 (100.0%) | 203 (84.6%) | **<0.001** |
|  |  | Yes | 0 (0.0%) | 37 (15.4%) |  |
|  | sFlt-1/PlGF | No/Unclear | 67 (100.0%) | 181 (75.4%) | **<0.001** |
|  |  | Yes | 0 (0.0%) | 59 (24.6%) |  |
|  | UtA-PI | No/Unclear | 41 (61.2%) | 103 (42.9%) | **0.008** |
|  |  | Yes | 26 (38.8%) | 137 (57.1%) |  |
| **Prophylactic treatments** | **Aspirin use** | No | 11 (16.4%) | 17 (7.1%) | **0.019** |
|  |  | Yes | 56 (83.6%) | 223 (92.9%) |  |
|  | **Individualized regimen** | No | 22 (39.3%) | 122 (54.7%) | **0.039** |
|  |  | Yes | 34 (60.7%) | 101 (45.3%) |  |
|  | **Aspirin Dose** | 100mg | 16 (72.7%) | 111 (91.0%) | **0.026*** |
|  |  | Others | 6 (27.3%) | 11 (9.0%) |  |
|  | **Aspirin frequency** | Once daily | 22 (100.0%) | 121 (99.2%) | 1.000* |
|  |  | Other frequencies | 0 (0.0%) | 1 (0.8%) |  |
|  | **Aspirin initiation** | 12 weeks of gestation | 6 (27.3%) | 46 (37.7%) | 0.348 |
|  |  | Others | 16 (72.7%) | 76 (62.3%) |  |
|  | **Aspirin discontinuation** | 36 weeks of gestation | 12 (54.5%) | 83 (68.0%) | 0.219 |
|  |  | others | 10 (45.5%) | 39 (32.0%) |  |
|  | **Factors influencing individualized** | | | | |
|  | maternal age | No | 10 (29.4%) | 41 (40.6%) | 0.245 |
|  |  | Yes | 24 (70.6%) | 60 (59.4%) |  |
|  | BMI/weight | No | 3 (8.8%) | 18 (17.8%) | 0.211 |
|  |  | Yes | 31 (91.2%) | 83 (82.2%) |  |
|  | multiple pregnancy | No | 16 (47.1%) | 71 (70.3%) | **0.014** |
|  |  | Yes | 18 (52.9%) | 30 (29.7%) |  |
|  | gestational age | No | 16 (47.1%) | 77 (76.2%) | **0.001** |
|  |  | Yes | 18 (52.9%) | 24 (23.8%) |  |
|  | hematologic disorders | No | 7 (20.6%) | 48 (47.5%) | **0.006** |
|  |  | Yes | 27 (79.4%) | 53 (52.5%) |  |
|  | **Regimen modification during treatment** | No | 41 (73.2%) | 185 (83.0%) | 0.096 |
|  |  | Yes | 15 (26.8%) | 38 (17.0%) |  |
|  | **Premature discontinuation** | No | 37 (66.1%) | 113 (50.7%) | **0.039** |
|  |  | Yes | 19 (33.9%) | 110 (49.3%) |  |
|  | **Concomitant use of calcium or heparin** | No | 44 (78.6%) | 192 (86.1%) | 0.163 |
|  |  | Yes | 12 (21.4%) | 31 (13.9%) |  |
| **Adherence to guidelines** | **Timing adherence** | No | 37 (56.9%) | 130 (59.9%) | 0.668 |
|  |  | Yes | 28 (43.1%) | 87 (40.1%) |  |
|  | **Risk factor adherence** | No | 11 (16.9%) | 34 (15.7%) | 0.809 |
|  |  | Yes | 54 (83.1%) | 183 (84.3%) |  |
|  | **Risk classification adherence** | No | 49 (75.4%) | 152 (70.0%) | 0.404 |
|  |  | Yes | 16 (24.6%) | 65 (30.0%) |  |
|  | **FMF model adherence** | No | 25 (64.1%) | 95 (63.3%) | 0.929 |
|  |  | Yes | 14 (35.9%) | 55 (36.7%) |  |
|  | **Aspirin indication adherence** | No | 43 (76.8%) | 170 (76.2%) | 0.931 |
|  |  | Yes | 13 (23.2%) | 53 (23.8%) |  |
|  | **Aspirin dose adherence** | No | 45 (67.2%) | 118 (49.2%) | **0.009** |
|  |  | Yes | 22 (32.8%) | 122 (50.8%) |  |
|  | **Aspirin initiation adherence** | No | 13 (59.1%) | 83 (68.0%) | 0.413 |
|  |  | Yes | 9 (40.9%) | 39 (32.0%) |  |
|  | **Aspirin discontinuation adherence** | No | 21 (95.5%) | 121 (99.2%) | 0.283* |
|  |  | Yes | 1 (4.5%) | 1 (0.8%) |  |
| ***Note***: Data are presented as n (%). *P* values were calculated using χ² test or Fisher’s exact test (*).  *P* values in bold indicated statistically significant differences between groups (*P* < 0.05). | | | | | |

**Table S3. Knowledge, practices, and adherence to clinical practice guidelines for pre-eclampsia risk management among obstetricians by age group (<40 vs ≥40 years)**

| **Module** | **Variable** | **Level** | **<40 years** | **≥40 years** | ***P* value** |
| --- | --- | --- | --- | --- | --- |
| **Risk prediction** | **Risk prediction** | No | 22 (12.4%) | 3 (2.3%) | **0.002** |
|  |  | Yes | 156 (87.6%) | 126 (97.7%) |  |
|  | **Timing of prediction** | | | | |
|  | first trimester | No | 31 (19.9%) | 18 (14.3%) | 0.218 |
|  |  | Yes | 125 (80.1%) | 108 (85.7%) |  |
|  | second trimester | No | 69 (44.2%) | 54 (42.9%) | 0.817 |
|  |  | Yes | 87 (55.8%) | 72 (57.1%) |  |
|  | third trimester | No | 68 (43.6%) | 42 (33.3%) | 0.079 |
|  |  | Yes | 88 (56.4%) | 84 (66.7%) |  |
|  | all three trimesters | No | 96 (61.5%) | 71 (56.3%) | 0.378 |
|  |  | Yes | 60 (38.5%) | 55 (43.7%) |  |
|  | **Risk factors used** | | | | |
|  | demographics | No | 17 (10.9%) | 12 (9.5%) | 0.706 |
|  |  | Yes | 139 (89.1%) | 114 (90.5%) |  |
|  | past medical history | No | 2 (1.3%) | 0 (0.0%) | 0.504* |
|  |  | Yes | 154 (98.7%) | 126 (100.0%) |  |
|  | past obstetric history | No | 1 (0.6%) | 1 (0.8%) | 1.000* |
|  |  | Yes | 155 (99.4%) | 125 (99.2%) |  |
|  | family history | No | 10 (6.4%) | 5 (4.0%) | 0.364 |
|  |  | Yes | 146 (93.6%) | 121 (96.0%) |  |
|  | current pregnancy | No | 3 (1.9%) | 2 (1.6%) | 1.000* |
|  |  | Yes | 153 (98.1%) | 124 (98.4%) |  |
|  | all five categories | No | 27 (17.3%) | 18 (14.3%) | 0.491 |
|  |  | Yes | 129 (82.7%) | 108 (85.7%) |  |
|  | **Risk classification** | Binary (low/high) | 46 (29.5%) | 35 (27.8%) | 0.752 |
|  |  | Ternary (low/medium/high) | 110 (70.5%) | 91 (72.2%) |  |
|  | **Awareness of FMF model** | No | 79 (44.4%) | 39 (30.2%) | **0.012** |
|  |  | Yes | 99 (55.6%) | 90 (69.8%) |  |
|  | **Use of FMF model** | No | 63 (63.6%) | 57 (63.3%) | 0.966 |
|  |  | Yes | 36 (36.4%) | 33 (36.7%) |  |
|  | **Barriers to FMF model implementation** | | | | |
|  | limited biomarker availability | No | 24 (38.1%) | 29 (50.9%) | 0.159 |
|  |  | Yes | 39 (61.9%) | 28 (49.1%) |  |
|  | no automatic calculation tools | No | 32 (50.8%) | 23 (40.4%) | 0.252 |
|  |  | Yes | 31 (49.2%) | 34 (59.6%) |  |
|  | no mandatory | No | 27 (42.9%) | 29 (50.9%) | 0.379 |
|  |  | Yes | 36 (57.1%) | 28 (49.1%) |  |
|  | cost | No | 40 (63.5%) | 42 (73.7%) | 0.231 |
|  |  | Yes | 23 (36.5%) | 15 (26.3%) |  |
|  | no perceived benefit | No | 60 (95.2%) | 54 (94.7%) | 1.000* |
|  |  | Yes | 3 (4.8%) | 3 (5.3%) |  |
|  | **Biomarker availability** | | | | |
|  | PlGF | No/Unclear | 126 (70.8%) | 86 (66.7%) | 0.441 |
|  |  | Yes | 52 (29.2%) | 43 (33.3%) |  |
|  | PAPP-A | No/Unclear | 151 (84.8%) | 103 (79.8%) | 0.254 |
|  |  | Yes | 27 (15.2%) | 26 (20.2%) |  |
|  | sFlt-1 | No/Unclear | 157 (88.2%) | 113 (87.6%) | 0.872 |
|  |  | Yes | 21 (11.8%) | 16 (12.4%) |  |
|  | sFlt-1/PlGF | No/Unclear | 142 (79.8%) | 106 (82.2%) | 0.599 |
|  |  | Yes | 36 (20.2%) | 23 (17.8%) |  |
|  | UtA-PI | No/Unclear | 85 (47.8%) | 59 (45.7%) | 0.727 |
|  |  | Yes | 93 (52.2%) | 70 (54.3%) |  |
| **Prophylactic treatments** | **Aspirin use** | No | 20 (11.2%) | 8 (6.2%) | 0.130 |
|  |  | Yes | 158 (88.8%) | 121 (93.8%) |  |
|  | **Individualized regimen** | No | 84 (53.2%) | 60 (49.6%) | 0.553 |
|  |  | Yes | 74 (46.8%) | 61 (50.4%) |  |
|  | **Aspirin Dose** | 100mg | 74 (88.1%) | 53 (88.3%) | 0.965 |
|  |  | Others | 10 (11.9%) | 7 (11.7%) |  |
|  | **Aspirin frequency** | Once daily | 83 (98.8%) | 60 (100.0%) | 1.000* |
|  |  | Other frequencies | 1 (1.2%) | 0 (0.0%) |  |
|  | **Aspirin initiation** | 12 weeks of gestation | 32 (38.1%) | 20 (33.3%) | 0.558 |
|  |  | Others | 52 (61.9%) | 40 (66.7%) |  |
|  | **Aspirin discontinuation** | 36 weeks of gestation | 54 (64.3%) | 41 (68.3%) | 0.613 |
|  |  | others | 30 (35.7%) | 9 (31.7%) |  |
|  | **Factors influencing individualized** | | | | |
|  | maternal age | No | 26 (35.1%) | 25 (41%) | 0.485 |
|  |  | Yes | 48 (64.9%) | 36 (59%) |  |
|  | BMI/weight | No | 14 (18.9%) | 7 (11.5%) | 0.235 |
|  |  | Yes | 60 (81.1%) | 54 (88.5%) |  |
|  | multiple pregnancy | No | 48 (64.9%) | 39 (63.9%) | 0.911 |
|  |  | Yes | 26 (35.1%) | 22 (36.1%) |  |
|  | gestational age | No | 52 (70.3%) | 41 (67.2%) | 0.703 |
|  |  | Yes | 22 (29.7%) | 20 (32.8%) |  |
|  | hematologic disorders | No | 26 (35.1%) | 29 (47.5%) | 0.144 |
|  |  | Yes | 48 (64.9%) | 32 (52.5%) |  |
|  | **Regimen modification during treatment** | No | 124 (78.5%) | 102 (84.3%) | 0.220 |
|  |  | Yes | 34 (21.5%) | 19 (15.7%) |  |
|  | **Premature discontinuation** | No | 82 (51.9%) | 68 (56.2%) | 0.475 |
|  |  | Yes | 76 (48.1%) | 53 (43.8%) |  |
|  | **Concomitant use of calcium or heparin** | No | 138 (87.3%) | 98 (81.0%) | 0.145 |
|  |  | Yes | 20 (12.7%) | 23 (19.0%) |  |
| **Adherence to guidelines** | **Timing adherence** | No | 96 (61.5%) | 71 (56.3%) | 0.378 |
|  |  | Yes | 60 (38.5%) | 55 (43.7%) |  |
|  | **Risk factor adherence** | No | 27 (17.3%) | 18 (14.3%) | 0.491 |
|  |  | Yes | 129 (82.7%) | 108 (85.7%) |  |
|  | **Risk classification adherence** | No | 110 (70.5%) | 91 (72.2%) | 0.752 |
|  |  | Yes | 46 (29.5%) | 35 (27.8%) |  |
|  | **FMF model adherence** | No | 63 (63.6%) | 57 (63.3%) | 0.966 |
|  |  | Yes | 36 (36.4%) | 33 (36.7%) |  |
|  | **Aspirin indication adherence** | No | 120 (75.9%) | 93 (76.9%) | 0.859 |
|  |  | Yes | 38 (24.1%) | 28 (23.1%) |  |
|  | **Aspirin dose adherence** | No | 94 (52.8%) | 69 (53.5%) | 0.906 |
|  |  | Yes | 84 (47.2%) | 60 (46.5%) |  |
|  | **Aspirin initiation adherence** | No | 55 (65.5%) | 41 (68.3%) | 0.720 |
|  |  | Yes | 29 (34.5%) | 19 (31.7%) |  |
|  | **Aspirin discontinuation adherence** | No | 82 (97.6%) | 60 (100.0%) | 0.510* |
|  |  | Yes | 2 (2.4%) | 0 (0.0%) |  |
| ***Note***: Data are presented as n (%). *P* values were calculated using χ² test or Fisher’s exact test (*).  *P* values in bold indicated statistically significant differences between groups (*P* < 0.05). | | | | | |

**Table S4. Knowledge, practices, and adherence to clinical practice guidelines for pre-eclampsia risk management among obstetricians by education level (Bachelor’s degree or below vs Master’s degree or above)**

| **Module** | **Variable** | **Level** | **Bachelor’s degree or below** | **Master’s degree or above** | ***P* value** |
| --- | --- | --- | --- | --- | --- |
| **Risk prediction** | **Risk prediction** | No | 9 (5.4%) | 16 (11.5%) | 0.050 |
|  |  | Yes | 159 (94.6%) | 123 (88.5%) |  |
|  | **Timing of prediction** | | | | |
|  | first trimester | No | 34 (21.4%) | 15 (12.2%) | **0.043** |
|  |  | Yes | 125 (78.6%) | 108 (87.8%) |  |
|  | second trimester | No | 59 (37.1%) | 64 (52.0%) | **0.012** |
|  |  | Yes | 100 (62.9%) | 59 (48.0%) |  |
|  | third trimester | No | 48 (30.2%) | 62 (50.4%) | **<0.001** |
|  |  | Yes | 111 (69.8%) | 61 (49.6%) |  |
|  | all three trimesters | No | 88 (55.3%) | 79 (64.2%) | 0.132 |
|  |  | Yes | 71 (44.7%) | 44 (35.8%) |  |
|  | **Risk factors used** | | | | |
|  | demographics | No | 20 (12.6%) | 9 (7.3%) | 0.149 |
|  |  | Yes | 139 (87.4%) | 114 (92.7%) |  |
|  | past medical history | No | 0 (0.0%) | 2 (1.6%) | 0.189* |
|  |  | Yes | 159 (100.0%) | 121 (98.4%) |  |
|  | past obstetric history | No | 0 (0.0%) | 2 (1.6%) | 0.189* |
|  |  | Yes | 159 (100.0%) | 121 (98.4%) |  |
|  | family history | No | 4 (2.5%) | 11 (8.9%) | **0.017** |
|  |  | Yes | 155 (97.5%) | 112 (91.1%) |  |
|  | current pregnancy | No | 3 (1.9%) | 2 (1.6%) | 1.000* |
|  |  | Yes | 156 (98.1%) | 121 (98.4%) |  |
|  | all five categories | No | 25 (15.7%) | 20 (16.3%) | 0.903 |
|  |  | Yes | 134 (84.3%) | 103 (83.7%) |  |
|  | **Risk classification** | Binary (low/high) | 46 (28.9%) | 35 (28.5%) | 0.930 |
|  |  | Ternary (low/medium/high) | 113 (71.1%) | 88 (71.5%) |  |
|  | **Awareness of FMF model** | No | 68 (40.5%) | 50 (36.0%) | 0.419 |
|  |  | Yes | 100 (59.5%) | 89 (64.0%) |  |
|  | **Use of FMF model** | No | 62 (62.0%) | 58 (65.2%) | 0.652 |
|  |  | Yes | 38 (38.0%) | 31 (34.8%) |  |
|  | **Barriers to FMF model implementation** | | | | |
|  | limited biomarker availability | No | 22 (35.5%) | 31 (53.4%) | **0.048** |
|  |  | Yes | 40 (64.5%) | 27 (46.6%) |  |
|  | no automatic calculation tools | No | 29 (46.8%) | 26 (44.8%) | 0.831 |
|  |  | Yes | 33 (53.2%) | 32 (55.2%) |  |
|  | no mandatory | No | 23 (37.1%) | 33 (56.9%) | **0.030** |
|  |  | Yes | 39 (62.9%) | 25 (43.1%) |  |
|  | cost | No | 41 (66.1%) | 41 (70.7%) | 0.591 |
|  |  | Yes | 21 (33.9%) | 17 (29.3%) |  |
|  | no perceived benefit | No | 61 (98.4%) | 53 (91.4%) | 0.106* |
|  |  | Yes | 1 (1.6%) | 5 (8.6%) |  |
|  | **Biomarker availability** | | | | |
|  | PlGF | No/Unclear | 141 (83.9%) | 71 (51.1%) | **<0.001** |
|  |  | Yes | 27 (16.1%) | 68 (48.9%) |  |
|  | PAPP-A | No/Unclear | 151 (89.9%) | 103 (74.1%) | **<0.001** |
|  |  | Yes | 17 (10.1%) | 36 (25.9%) |  |
|  | sFlt-1 | No/Unclear | 164 (97.6%) | 106 (76.3%) | **<0.001** |
|  |  | Yes | 4 (2.4%) | 33 (23.7%) |  |
|  | sFlt-1/PlGF | No/Unclear | 159 (94.6%) | 89 (64.0%) | **<0.001** |
|  |  | Yes | 9 (5.4%) | 50 (36.0%) |  |
|  | UtA-PI | No/Unclear | 88 (52.4%) | 56 (40.3%) | **0.035** |
|  |  | Yes | 80 (47.6%) | 83 (59.7%) |  |
| **Prophylactic treatments** | **Aspirin use** | No | 17 (10.1%) | 11 (7.9%) | 0.504 |
|  |  | Yes | 151 (89.9%) | 128 (92.1%) |  |
|  | **Individualized regimen** | No | 84 (55.6%) | 60 (46.9%) | 0.145 |
|  |  | Yes | 67 (44.4%) | 68 (53.1%) |  |
|  | **Aspirin Dose** | 100mg | 75 (89.3%) | 52 (86.7%) | 0.631 |
|  |  | Others | 9 (10.7%) | 8 (13.3%) |  |
|  | **Aspirin frequency** | Once daily | 83 (98.8%) | 60 (100.0%) | 1.000* |
|  |  | Other frequencies | 1 (1.2%) | 0 (0.0%) |  |
|  | **Aspirin initiation** | 12 weeks of gestation | 29 (34.5%) | 23 (38.3%) | 0.639 |
|  |  | Others | 55 (65.5%) | 37 (61.7%) |  |
|  | **Aspirin discontinuation** | 36 weeks of gestation | 55 (65.5%) | 40 (66.7%) | 0.882 |
|  |  | others | 29 (34.5%) | 20 (33.3%) |  |
|  | **Factors influencing individualized** | | | | |
|  | maternal age | No | 20 (29.9%) | 31 (45.6%) | 0.059 |
|  |  | Yes | 47 (70.1%) | 37 (54.4%) |  |
|  | BMI/weight | No | 9 (13.4%) | 12 (17.6%) | 0.499 |
|  |  | Yes | 58 (86.6%) | 56 (82.4%) |  |
|  | multiple pregnancy | No | 41 (61.2%) | 46 (67.6%) | 0.434 |
|  |  | Yes | 26 (38.8%) | 22 (32.4%) |  |
|  | gestational age | No | 39 (58.2%) | 54 (79.4%) | **0.008** |
|  |  | Yes | 28 (41.8%) | 14 (20.6%) |  |
|  | hematologic disorders | No | 20 (29.9%) | 35 (51.5%) | **0.011** |
|  |  | Yes | 47 (70.1%) | 33 (48.5%) |  |
|  | **Regimen modification during treatment** | No | 124 (82.1%) | 102 (79.7%) | 0.606 |
|  |  | Yes | 27 (17.9%) | 26 (20.3%) |  |
|  | **Premature discontinuation** | No | 89 (58.9%) | 61 (47.7%) | 0.060 |
|  |  | Yes | 62 (41.1%) | 67 (52.3%) |  |
|  | **Concomitant use of calcium or heparin** | No | 126 (83.4%) | 110 (85.9%) | 0.565 |
|  |  | Yes | 25 (16.6%) | 18 (14.1%) |  |
| **Adherence to guidelines** | **Timing adherence** | No | 88 (55.3%) | 79 (64.2%) | 0.132 |
|  |  | Yes | 71 (44.7%) | 44 (35.8%) |  |
|  | **Risk factor adherence** | No | 25 (15.7%) | 20 (16.3%) | 0.903 |
|  |  | Yes | 134 (84.3%) | 103 (83.7%) |  |
|  | **Risk classification adherence** | No | 113 (71.1%) | 88 (71.5%) | 0.930 |
|  |  | Yes | 46 (28.9%) | 35 (28.5%) |  |
|  | **FMF model adherence** | No | 62 (62.0%) | 58 (65.2%) | 0.652 |
|  |  | Yes | 38 (38.0%) | 31 (34.8%) |  |
|  | **Aspirin indication adherence** | No | 119 (78.8%) | 94 (73.4%) | 0.293 |
|  |  | Yes | 32 (21.2%) | 34 (26.6%) |  |
|  | **Aspirin dose adherence** | No | 84 (50.0%) | 79 (56.8%) | 0.232 |
|  |  | Yes | 84 (50.0%) | 60 (43.2%) |  |
|  | **Aspirin initiation adherence** | No | 58 (69.0%) | 38 (63.3%) | 0.473 |
|  |  | Yes | 26 (31.0%) | 22 (36.7%) |  |
|  | **Aspirin discontinuation adherence** | No | 82 (97.6%) | 60 (100.0%) | 0.510* |
|  |  | Yes | 2 (2.4%) | 0 (0.0%) |  |
| ***Note***: Data are presented as n (%). *P* values were calculated using χ² test or Fisher’s exact test (*).  *P* values in bold indicated statistically significant differences between groups (*P* < 0.05). | | | | | |

**Table S5. Knowledge, practices, and adherence to clinical practice guidelines for pre-eclampsia risk management among obstetricians by professional title (Intermediate or below vs Senior)**

| **Module** | **Variable** | **Level** | **Intermediate or below** | **Senior** | ***P* value** |
| --- | --- | --- | --- | --- | --- |
| **Risk prediction** | **Risk prediction** | No | 21 (10.7%) | 4 (3.6%) | **0.031** |
|  |  | Yes | 176 (89.3%) | 106 (96.4%) |  |
|  | **Timing of prediction** | | | | |
|  | first trimester | No | 40 (22.7%) | 9 (8.5%) | **0.002** |
|  |  | Yes | 136 (77.3%) | 97 (91.5%) |  |
|  | second trimester | No | 74 (42.0%) | 49 (46.2%) | 0.493 |
|  |  | Yes | 102 (58.0%) | 57 (53.8%) |  |
|  | third trimester | No | 66 (37.5%) | 44 (41.5%) | 0.504 |
|  |  | Yes | 110 (62.5%) | 62 (58.5%) |  |
|  | all three trimesters | No | 103 (58.5%) | 64 (60.4%) | 0.759 |
|  |  | Yes | 73 (41.5%) | 42 (39.6%) |  |
|  | **Risk factors used** | | | | |
|  | demographics | No | 19 (10.8%) | 10 (9.4%) | 0.715 |
|  |  | Yes | 157 (89.2%) | 96 (90.6%) |  |
|  | past medical history | No | 2 (1.1%) | 0 (0.0%) | 0.529* |
|  |  | Yes | 174 (98.9%) | 106 (100.0%) |  |
|  | past obstetric history | No | 1 (0.6%) | 1 (0.9%) | 1.000* |
|  |  | Yes | 175 (99.4%) | 105 (99.1%) |  |
|  | family history | No | 8 (4.5%) | 7 (6.6%) | 0.456 |
|  |  | Yes | 168 (95.5%) | 99 (93.4%) |  |
|  | current pregnancy | No | 4 (2.3%) | 1 (0.9%) | 0.653* |
|  |  | Yes | 172 (97.7%) | 105 (99.1%) |  |
|  | all five categories | No | 28 (15.9%) | 17 (16.0%) | 0.977 |
|  |  | Yes | 148 (84.1%) | 89 (84.0%) |  |
|  | **Risk classification** | Binary (low/high) | 45 (25.6%) | 36 (34.0%) | 0.131 |
|  |  | Ternary (low/medium/high) | 131 (74.4%) | 70 (66.0%) |  |
|  | **Awareness of FMF model** | No | 82 (41.6%) | 36 (32.7%) | 0.124 |
|  |  | Yes | 115 (58.4%) | 74 (67.3%) |  |
|  | **Use of FMF model** | No | 69 (60%) | 51 (68.9%) | 0.214 |
|  |  | Yes | 46 (40%) | 23 (31.1%) |  |
|  | **Barriers to FMF model implementation** | | | | |
|  | limited biomarker availability | No | 25 (36.2%) | 28 (54.9%) | **0.042** |
|  |  | Yes | 44 (63.8%) | 23 (45.1%) |  |
|  | no automatic calculation tools | No | 36 (52.2%) | 19 (37.3%) | 0.105 |
|  |  | Yes | 33 (47.8%) | 32 (62.7%) |  |
|  | no mandatory | No | 35 (50.7%) | 21 (41.2%) | 0.300 |
|  |  | Yes | 34 (49.3%) | 30 (58.8%) |  |
|  | cost | No | 45 (65.2%) | 37 (72.5%) | 0.393 |
|  |  | Yes | 24 (34.8%) | 14 (27.5%) |  |
|  | no perceived benefit | No | 66 (95.7%) | 48 (94.1%) | 0.698* |
|  |  | Yes | 3 (4.3%) | 3 (5.9%) |  |
|  | **Biomarker availability** | | | | |
|  | PlGF | No/Unclear | 137 (69.5%) | 75 (68.2%) | 0.805 |
|  |  | Yes | 60 (30.5%) | 35 (31.8%) |  |
|  | PAPP-A | No/Unclear | 165 (83.8%) | 89 (80.9%) | 0.527 |
|  |  | Yes | 32 (16.2%) | 21 (19.1%) |  |
|  | sFlt-1 | No/Unclear | 172 (87.3%) | 98 (89.1%) | 0.646 |
|  |  | Yes | 25 (12.7%) | 12 (10.9%) |  |
|  | sFlt-1/PlGF | No/Unclear | 158 (80.2%) | 90 (81.8%) | 0.731 |
|  |  | Yes | 39 (19.8%) | 20 (18.2%) |  |
|  | UtA-PI | No/Unclear | 93 (47.2%) | 51 (46.4%) | 0.887 |
|  |  | Yes | 104 (52.8%) | 59 (53.6%) |  |
| **Prophylactic treatments** | **Aspirin use** | No | 25 (12.7%) | 3 (2.7%) | **0.004** |
|  |  | Yes | 172 (87.3%) | 107 (97.3%) |  |
|  | **Individualized regimen** | No | 82 (47.7%) | 62 (57.9%) | 0.095 |
|  |  | Yes | 90 (52.3%) | 45 (42.1%) |  |
|  | **Aspirin Dose** | 100mg | 70 (85.4%) | 57 (91.9%) | 0.226 |
|  |  | Others | 12 (14.6%) | 5 (8.1%) |  |
|  | **Aspirin frequency** | Once daily | 81 (98.8%) | 62 (100.0%) | 1.000* |
|  |  | Other frequencies | 1 (1.2%) | 0 (0.0%) |  |
|  | **Aspirin initiation** | 12 weeks of gestation | 32 (39.0%) | 20 (32.3%) | 0.403 |
|  |  | Others | 50 (61.0%) | 42 (67.7%) |  |
|  | **Aspirin discontinuation** | 36 weeks of gestation | 55 (67.1%) | 40 (64.5%) | 0.748 |
|  |  | others | 27 (32.9%) | 22 (35.5%) |  |
|  | **Factors influencing individualized** | | | | |
|  | maternal age | No | 30 (33.3%) | 21 (46.7%) | 0.132 |
|  |  | Yes | 60 (66.7%) | 24 (53.3%) |  |
|  | BMI/weight | No | 15 (16.7%) | 6 (13.3%) | 0.614 |
|  |  | Yes | 75 (83.3%) | 39 (86.7%) |  |
|  | multiple pregnancy | No | 55 (61.1%) | 32 (71.1%) | 0.253 |
|  |  | Yes | 35 (38.9%) | 13 (28.9%) |  |
|  | gestational age | No | 61 (67.8%) | 32 (71.1%) | 0.693 |
|  |  | Yes | 29 (32.2%) | 13 (28.9%) |  |
|  | hematologic disorders | No | 34 (37.8%) | 21 (46.7%) | 0.322 |
|  |  | Yes | 56 (62.2%) | 24 (53.3%) |  |
|  | **Regimen modification during treatment** | No | 135 (78.5%) | 91 (85.0%) | 0.175 |
|  |  | Yes | 37 (21.5%) | 16 (15.0%) |  |
|  | **Premature discontinuation** | No | 91 (52.9%) | 59 (55.1%) | 0.716 |
|  |  | Yes | 81 (47.1%) | 48 (44.9%) |  |
|  | **Concomitant use of calcium or heparin** | No | 154 (89.5%) | 82 (76.6%) | **0.004** |
|  |  | Yes | 18 (10.5%) | 25 (23.4%) |  |
| **Adherence to guidelines** | **Timing adherence** | No | 103 (58.5%) | 64 (60.4%) | 0.759 |
|  |  | Yes | 73 (41.5%) | 42 (39.6%) |  |
|  | **Risk factor adherence** | No | 28 (15.9%) | 17 (16.0%) | 0.977 |
|  |  | Yes | 148 (84.1%) | 89 (84.0%) |  |
|  | **Risk classification adherence** | No | 131 (74.4%) | 70 (66.0%) | 0.131 |
|  |  | Yes | 45 (25.6%) | 36 (34.0%) |  |
|  | **FMF model adherence** | No | 69 (60.0%) | 51 (68.9%) | 0.214 |
|  |  | Yes | 46 (40.0%) | 23 (31.1%) |  |
|  | **Aspirin indication adherence** | No | 130 (75.6%) | 83 (77.6%) | 0.704 |
|  |  | Yes | 42 (24.4%) | 24 (22.4%) |  |
|  | **Aspirin dose adherence** | No | 115 (58.4%) | 48 (43.6%) | **0.013** |
|  |  | Yes | 82 (41.6%) | 62 (56.4%) |  |
|  | **Aspirin initiation adherence** | No | 56 (68.3%) | 40 (64.5%) | 0.634 |
|  |  | Yes | 26 (31.7%) | 22 (35.5%) |  |
|  | **Aspirin discontinuation adherence** | No | 80 (97.6%) | 62 (100.0%) | 0.506* |
|  |  | Yes | 2 (2.4%) | 0 (0.0%) |  |
| ***Note***: Data are presented as n (%). *P* values were calculated using χ² test or Fisher’s exact test (*).  *P* values in bold indicated statistically significant differences between groups (*P* < 0.05). | | | | | |

**Table S6. Knowledge, practices, and adherence to clinical practice guidelines for pre-eclampsia risk management among obstetricians by years of experience (<10 vs ≥10 years)**

| **Module** | **Variable** | **Level** | **<10 years** | **≥10 years** | ***P* value** |
| --- | --- | --- | --- | --- | --- |
| **Risk prediction** | **Risk prediction** | No | 18 (16.4%) | 7 (3.6%) | **<0.001** |
|  |  | Yes | 92 (83.6%) | 190 (96.4%) |  |
|  | **Timing of prediction** | | | | |
|  | first trimester | No | 26 (28.3%) | 23 (12.1%) | **<0.001** |
|  |  | Yes | 66 (71.7%) | 167 (87.9%) |  |
|  | second trimester | No | 41 (44.6%) | 82 (43.2%) | 0.823 |
|  |  | Yes | 51 (55.4%) | 108 (56.8%) |  |
|  | third trimester | No | 36 (39.1%) | 74 (38.9%) | 0.976 |
|  |  | Yes | 56 (60.9%) | 116 (61.1%) |  |
|  | all three trimesters | No | 56 (60.9%) | 111 (58.4%) | 0.695 |
|  |  | Yes | 36 (39.1%) | 79 (41.6%) |  |
|  | **Risk factors used** | | | | |
|  | demographics | No | 9 (9.8%) | 20 (10.5%) | 0.847 |
|  |  | Yes | 83 (90.2%) | 170 (89.5%) |  |
|  | past medical history | No | 2 (2.2%) | 0 (0.0%) | 0.106* |
|  |  | Yes | 90 (97.8%) | 190 (100.0%) |  |
|  | past obstetric history | No | 1 (1.1%) | 1 (0.5%) | 0.547* |
|  |  | Yes | 1 (98.9%) | 189 (99.5%) |  |
|  | family history | No | 6 (6.5%) | 9 (4.7%) | 0.576* |
|  |  | Yes | 86 (93.5%) | 181 (95.3%) |  |
|  | current pregnancy | No | 1 (1.1%) | 4 (2.1%) | 1.000* |
|  |  | Yes | 91 (98.9%) | 186 (97.9%) |  |
|  | all five categories | No | 14 (15.2%) | 31 (16.3%) | 0.813 |
|  |  | Yes | 78 (84.8%) | 159 (83.7%) |  |
|  | **Risk classification** | Binary (low/high) | 27 (29.3%) | 54 (28.4%) | 0.872 |
|  |  | Ternary (low/medium/high) | 65 (70.7%) | 136 (71.6%) |  |
|  | **Awareness of FMF model** | No | 51 (46.4%) | 67 (34.0%) | **0.033** |
|  |  | Yes | 59 (53.6%) | 130 (66.0%) |  |
|  | **Use of FMF model** | No | 36 (61.0%) | 84 (64.6%) | 0.634 |
|  |  | Yes | 23 (39.0%) | 46 (35.4%) |  |
|  | **Barriers to FMF model implementation** | | | | |
|  | limited biomarker availability | No | 18 (50.0%) | 35 (41.7%) | 0.400 |
|  |  | Yes | 18 (50.0%) | 49 (58.3%) |  |
|  | no automatic calculation tools | No | 21 (58.3%) | 34 (40.5%) | 0.072 |
|  |  | Yes | 15 (41.7%) | 50 (59.5%) |  |
|  | no mandatory | No | 15 (41.7%) | 41 (48.8%) | 0.472 |
|  |  | Yes | 21 (58.3%) | 43 (51.2%) |  |
|  | cost | No | 24 (66.7%) | 58 (69.0%) | 0.797 |
|  |  | Yes | 12 (33.3%) | 26 (31.0%) |  |
|  | no perceived benefit | No | 34 (94.4%) | 80 (95.2%) | 1.000* |
|  |  | Yes | 2 (5.6%) | 4 (4.8%) |  |
|  | **Biomarker availability** | | | | |
|  | PlGF | No/Unclear | 76 (69.1%) | 136 (69.0%) | 0.992 |
|  |  | Yes | 34 (30.9%) | 61 (31.0%) |  |
|  | PAPP-A | No/Unclear | 96 (87.3%) | 158 (80.2%) | 0.116 |
|  |  | Yes | 14 (12.7%) | 39 (19.8%) |  |
|  | sFlt-1 | No/Unclear | 95 (86.4%) | 175 (88.8%) | 0.524 |
|  |  | Yes | 15 (13.6%) | 22 (11.2%) |  |
|  | sFlt-1/PlGF | No/Unclear | 86 (78.2%) | 162 (82.2%) | 0.388 |
|  |  | Yes | 24 (21.8%) | 35 (17.8%) |  |
|  | UtA-PI | No/Unclear | 57 (51.8%) | 87 (44.2%) | 0.197 |
|  |  | Yes | 53 (48.2%) | 110 (55.8%) |  |
| **Prophylactic treatments** | **Aspirin use** | No | 19 (17.3%) | 9 (4.6%) | **<0.001** |
|  |  | Yes | 91 (82.7%) | 188 (95.4%) |  |
|  | **Individualized regimen** | No | 38 (41.8%) | 106 (56.4%) | **0.022** |
|  |  | Yes | 53 (58.2%) | 82 (43.6%) |  |
|  | **Aspirin Dose** | 100mg | 32 (84.2%) | 95 (89.6%) | 0.388* |
|  |  | Others | 6 (15.8%) | 11 (10.4%) |  |
|  | **Aspirin frequency** | Once daily | 37 (97.4%) | 106 (100.0%) | 0.264* |
|  |  | Other frequencies | 1 (2.6%) | 0 (0.0%) |  |
|  | **Aspirin initiation** | 12 weeks of gestation | 12 (31.6%) | 40 (37.7%) | 0.498 |
|  |  | Others | 26 (68.4%) | 66 (62.3%) |  |
|  | **Aspirin discontinuation** | 36 weeks of gestation | 25 (65.8%) | 70 (66.0%) | 0.978 |
|  |  | others | 13 (34.2%) | 36 (34.0%) |  |
|  | **Factors influencing individualized** | | | | |
|  | maternal age | No | 16 (30.2%) | 35 (42.7%) | 0.144 |
|  |  | Yes | 37 (69.8%) | 47 (57.3%) |  |
|  | BMI/weight | No | 9 (17.0%) | 12 (14.6%) | 0.713 |
|  |  | Yes | 44 (83.0%) | 70 (85.4%) |  |
|  | multiple pregnancy | No | 35 (66.0%) | 52 (63.4%) | 0.756 |
|  |  | Yes | 18 (34.0%) | 30 (36.6%) |  |
|  | gestational age | No | 36 (67.9%) | 57 (69.5%) | 0.846 |
|  |  | Yes | 17 (32.1%) | 25 (30.5%) |  |
|  | hematologic disorders | No | 19 (35.8%) | 36 (43.9%) | 0.352 |
|  |  | Yes | 34 (64.2%) | 46 (56.1%) |  |
|  | **Regimen modification during treatment** | No | 67 (73.6%) | 159 (84.6%) | **0.029** |
|  |  | Yes | 24 (26.4%) | 29 (15.4%) |  |
|  | **Premature discontinuation** | No | 46 (50.5%) | 104 (55.3%) | 0.454 |
|  |  | Yes | 45 (49.5%) | 84 (44.7%) |  |
|  | **Concomitant use of calcium or heparin** | No | 82 (90.1%) | 154 (81.9%) | 0.076 |
|  |  | Yes | 9 (9.9%) | 34 (18.1%) |  |
| **Adherence to guidelines** | **Timing adherence** | No | 56 (60.9%) | 111 (58.4%) | 0.695 |
|  |  | Yes | 36 (39.1%) | 79 (41.6%) |  |
|  | **Risk factor adherence** | No | 14 (15.2%) | 31 (16.3%) | 0.813 |
|  |  | Yes | 78 (84.8%) | 159 (83.7%) |  |
|  | **Risk classification adherence** | No | 65 (70.7%) | 136 (71.6%) | 0.872 |
|  |  | Yes | 27 (29.3%) | 54 (28.4%) |  |
|  | **FMF model adherence** | No | 36 (61.0%) | 84 (64.6%) | 0.634 |
|  |  | Yes | 23 (39.0%) | 46 (35.4%) |  |
|  | **Aspirin indication adherence** | No | 67 (73.6%) | 146 (77.7%) | 0.457 |
|  |  | Yes | 24 (26.4%) | 42 (22.3%) |  |
|  | **Aspirin dose adherence** | No | 72 (65.5%) | 91 (46.2%) | **0.001** |
|  |  | Yes | 38 (34.5%) | 106 (53.8%) |  |
|  | **Aspirin initiation adherence** | No | 25 (65.8%) | 71 (67.0%) | 0.894 |
|  |  | Yes | 13 (34.2%) | 35 (33.0%) |  |
|  | **Aspirin discontinuation adherence** | No | 36 (94.7%) | 106(100.0%) | 0.068* |
|  |  | Yes | 2 (5.3%) | 0 (0.0%) |  |
| ***Note***: Data are presented as n (%). *P* values were calculated using χ² test or Fisher’s exact test (*).  *P* values in bold indicated statistically significant differences between groups (*P* < 0.05). | | | | | |
